# Modeling craniofacial development reveals spatiotemporal constraints on robust patterning of the mandibular arch

**DOI:** 10.1371/journal.pcbi.1006569

**Published:** 2018-11-27

**Authors:** Lina Meinecke, Praveer P. Sharma, Huijing Du, Lei Zhang, Qing Nie, Thomas F. Schilling

**Affiliations:** 1 Department of Mathematics, University of California, Irvine, CA, United States of America; 2 Center for Complex Biological Systems, University of California, Irvine, CA, United States of America; 3 Department of Developmental and Cell Biology, University of California, Irvine, CA, United States of America; 4 Department of Mathematics, University of Nebraska, Lincoln, NE, United States of America; 5 Beijing International Center for Mathematical Research, Peking University, Beijing, China; 6 Center for Quantitative Biology, Peking University, Beijing, China; Oxford, UNITED KINGDOM

## Abstract

How does pattern formation occur accurately when confronted with tissue growth and stochastic fluctuations (noise) in gene expression? Dorso-ventral (D-V) patterning of the mandibular arch specifies upper versus lower jaw skeletal elements through a combination of Bone morphogenetic protein (Bmp), Endothelin-1 (Edn1), and Notch signaling, and this system is highly robust. We combine NanoString experiments of early D-V gene expression with live imaging of arch development in zebrafish to construct a computational model of the D-V mandibular patterning network. The model recapitulates published genetic perturbations in arch development. Patterning is most sensitive to changes in Bmp signaling, and the temporal order of gene expression modulates the response of the patterning network to noise. Thus, our integrated systems biology approach reveals non-intuitive features of the complex signaling system crucial for craniofacial development, including novel insights into roles of gene expression timing and stochasticity in signaling and gene regulation.

## Author summary

Proper development of the body requires boundaries to form between regions in which cells will form different structures, and these boundaries need to be properly organized in space. This must occur accurately even in moving, dividing cells and in the presence of the noise that is inherent in all biochemical processes. We use development of the upper and lower jaw as a model to study boundary formation. In this work, we combine detailed experimental measurements with computational modeling to investigate the role the timing of gene expression plays in organizing spatial boundaries, and find that the different orders of gene expression navigate a tradeoff between precision and accuracy in boundary positioning.

## Introduction

A fundamental question in developmental biology is pattern formation, i.e. the acquisition of positional identity in cells resulting in spatially organized domains of gene expression. Computational analyses have long sought to address how patterning occurs in growing tissues that change their size and shape by modeling morphogen gradients, signaling between cells, geometric transformations and other mathematically-amenable aspects of development [1–4]. More recently, computational modeling has revealed how signaling networks integrate with one another and the importance of feedback loops in precise regulation of early developmental patterning systems [5–11]. Models for more complex developing structures such as vertebrate limb buds [12,13], hair follicles [14,15], pigment cells in the skin [16], the spinal cord [17,18] or the palate [19,20] require integrating multiple signals within rapidly expanding three-dimensional (3D) tissues.

Pharyngeal arches are bilateral, segmentally-repeated structures that form in the ventral head of vertebrate embryos and give rise to skeletal, muscle and connective tissues of the face and neck, including the upper and lower jaws. Arches are complex both in their 3D morphologies and in their embryonic cellular origins. Streams of cranial neural crest (NC) cells migrate into each arch segment and surround cores of myogenic/vasculogenic mesoderm. The surrounding ectodermal and endodermal epithelia produce signals that subsequently pattern the arch along its dorso-ventral (D-V) axis, resulting in at least three early domains: ventral (V), intermediate (I), and dorsal (D) [21–23]. D-V arch patterning involves a highly-conserved signaling network consisting of the Bone morphogenetic protein 2/4/7 (Bmp) and Endothelin-1 (Edn1) signaling pathways, secreted by ventral arch epithelia [24–28], and dorsal Jagged1 (Jag1)/Notch signaling [29,30]. Errors in these signals can lead to craniofacial birth defects, such as auriculocondylar syndrome in humans, in which Edn1 signal transduction is disrupted leading to partial homeotic transformation of ventral skeletal elements to a dorsal fate [31,32]. Understanding how D-V domains arise in the midst of NC migration and arch growth is both an experimental and a computational challenge.

Both Edn1 and Bmp are crucial for ventral and intermediate arch development, but with distinct effects on gene expression [24,27,30,33–35]. Bmp, which acts as a morphogen in many contexts [36–38], primarily induces and maintains genes expressed ventrally such as Hand2, a critical transcription factor for ventral mandibular identity. In contrast, while Edn1 also induces ventral genes initially in a concentration-dependent manner [26,39] it later becomes primarily required for expression of intermediate genes such as Dlx5/6, which are required for ventral/intermediate mandibular (lower jaw and jaw joint) development, and less dependent on Bmp [24,29,30]. Craniofacial patterning defects in *edn1*^*-/-*^ mutants can be largely rescued by injection of Edn1 protein throughout the arch [27], and recent work suggests Edn1 plays a more permissive than instructive role [40].

Given their common targets, what are the advantages of having these two ventral morphogens acting in parallel during early D-V arch patterning? In addition, how does patterning occur robustly in the face of continuous cell divisions, rearrangements, and noise in both the signaling molecules and their downstream gene regulatory networks (GRNs)? To address these questions, we have developed the first computational model of arch D-V patterning that incorporates growth, migration, gene expression and different sources of noise. We represent the known components of the arch GRN with a system of ordinary differential equations (ODEs) that accurately reproduces published genetic perturbations of arch D-V patterning. We establish the model using measurements of spatiotemporal patterns of gene expression and 3D NC cell movements obtained from time-lapse movies of live zebrafish embryos. Quantitative temporal gene expression data reveal that intermediate domain genes are expressed before genes marking the ventral domain and dorsal genes are expressed last. The model confirms that this temporal order of intermediate-ventral-dorsal (IVD) patterning improves some aspects of robustness of D-V patterning (precision, referring to consistency across simulations/embryos), while making other aspects (accuracy, referring to closeness to the theoretical ideal pattern) more sensitive. The model further suggests that Bmp signaling primarily establishes the sizes and positions of patterning domains, while Edn1 plays a permissive role, and that noise in the GRN and each of the signaling pathways affects patterning differently. Our model reveals novel features of the early spatiotemporal dynamics of gene expression that are critical for patterning the complex 3D structure of the craniofacial skeleton during embryogenesis.

## Materials and methods

### Ethics statement

Institutional Animal Care and Use Committee protocol #2000–2149.

### Biological experiments

#### Animals

*hand2*:*eGFP* [41], *dlx5a*:*eGFP* [42], *sox10*:*lyn-tdTomato* [43], *fli1a*:*eGFP* [44], and *sox10*:*dsRed* [45] transgenic zebrafish lines have been previously described.

#### Live imaging

Whole zebrafish embryos were mounted laterally in 0.5% low-melt agarose (Apex Bioresearch Products) inside glass-bottom microwell dishes (MatTek Corporation). Imaging was performed on a Nikon Eclipse Ti confocal microscope, with excitation by 488nm (eGFP) and 561nm (tdTomato and dsRed) lasers. All imaging was done at 28.5°C, with embryos staged as described previously [46].

#### Hybridization chain reaction (HCR) *in situ* hybridization

HCR was performed as described [47]. Briefly, this *in situ* protocol involves DNA probes that bind to target mRNA and subsequently trigger hybridization chain reactions in fluorescent-labeled hairpins that self-assemble into localized polymers. The amount of HCR signal is directly proportional to the amount of target mRNA, and thus provides a quantitative measure of gene expression, unlike chromatogenic *in situ* hybridization, while preserving spatial information in whole-mount embryos [48]. We used AB wild-type zebrafish embryos fixed overnight at 4°C in 4% paraformaldehyde. These were whole-mounted in low-melt agarose and imaged on a Nikon Eclipse Ti confocal microscope. Probes were ordered from Molecular Instruments for the following genes (accession numbers shown) and their hairpins were labeled with the indicated fluorophores: *dlx2a*: NM_131311.2, Alexa 647; *dlx3b*: NM_131322.2, Alexa 546; *dlx5a*: NM_131306.2, Alexa 546; *hand2*: NM_131626.3, Alexa 488.

#### NanoString

NanoString technology was used for direct quantification of mRNA transcripts [49]. Double-transgenic *fli1a*:*eGFP;sox10*:*dsRed* zebrafish embryos were dissociated as described previously [50]. Fluorescence-associated cell sorting (FACS) was performed on a BD Aria II sorter collecting only eGFP/dsRed double-positive cells. Cells were re-suspended in 1.5 μl buffer RLT (Qiagen) per 6500 cells, and delivered to the UC Irvine Genomics High Throughput Sequencing Facility for processing. Approximately 20–30 embryos were used for each biological replicate. The resulting NanoString data were processed using the nSolver Analysis Software 3.0 (Nanostring Technologies Inc.). The expression values were normalized using a panel of housekeeping genes and represent average per-cell values.

### Modeling

#### The one-dimensional (1D) model

**Fig 1:**
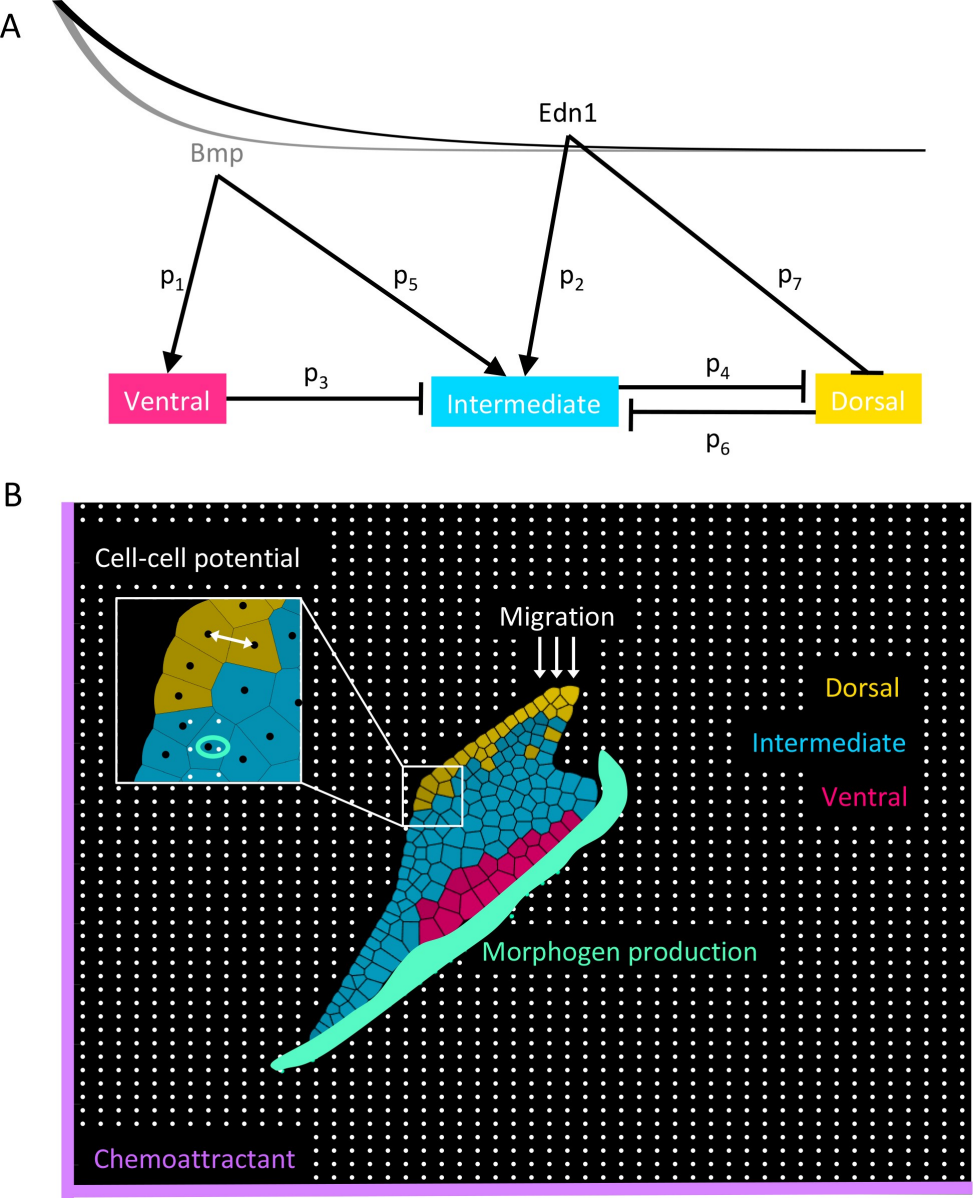
Modeling arch dorsal-ventral (D-V) patterning. (A) The gene regulatory network (GRN) used to compute the gene expression inside each cell. The lines represent the declining morphogen gradients from ventral to dorsal (Bmp left, Edn1 right). The morphogen gradients act as space-dependent inputs into the ODE system, specifying cells based on their locations along the D-V axis. The mathematical model is a system of coupled ordinary differential equations (ODEs) (Eqs 1–3), where gene interactions are modeled by Hill functions with the microscopic dissociation constants *p*_*1*_*-p*_*7*_ (S1 Table) and production and degradation parameters specified for each GRN component (S2 Table)_._ (B) A cell-based two-dimensional (2D) model. Cells are represented as vertices (black dots in inset) and a generalized Morse potential between these vertices (white arrow in inset) accounts for cell-cell adhesion and cell volume. Cell membranes are computed using Voronoi tessellation between nodes. The collective motion of cells is directed by the boundary conditions and a global chemoattractant pulling them ventrally. At each time step, migrating cells are added at random locations dorsally (white arrows), cells randomly divide, the morphogen production zone is updated (green area) and the quasi steady state of the morphogen gradients is computed on the discrete grid (white dots). Cells experience morphogen concentrations equal to that of the grid point closest to its cell center (green ring in inset). These values are *A*_*1*_*(x)* and *A*_*2*_*(x)* in Eqs 1–3 which are solved numerically for each individual cell. Cells are specified as ventral (pink), intermediate (cyan) or dorsal (yellow) if the respective gene expression exceeds 20% and is higher than all the other genes. Colors become darker gradually as the level of expression increases. Mechanical parameters are specified in S3 Table.

Since the embryonic mandibular arch is initially patterned into three domains along the D-V axis, we first create a 1D model to capture D-V patterning in a computationally efficient manner. To construct a minimal GRN (Fig 1A) we include published gene expression studies and genetic perturbations of arch development (S1–S3 Tables) [24,29,30,42,50]. Ventral (V) genes include *hand2*, intermediate (I) genes include *dlx3b/4a/4b/5a/6a* and dorsal (D) genes include *jag1* and *hey1*.

The two morphogens Bmp and Edn1 are secreted at the ventral end of the arch and form a declining gradient from ventral to dorsal. We model the two gradients as time-independent, exponentially declining functions from ventral to dorsal, solving the steady state with a source term at the ventral domain boundary and constant morphogen degradation. Measurements of diffusion coefficients for Edn1 range from 104.2–140.5 μm^2^/s [51,52], and for Bmps range from 2.0–4.4 μm^2^/s [53,54], suggesting that Edn1 diffuses farther dorsally than Bmps. Grem2, a Bmp antagonist expressed dorsally, also acts to restrict Bmp signalling to the ventral arch [30]. We thus extend the Edn1 gradient farther dorsally than the Bmp gradient (Fig 1A). Mathematically we represent the GRN by the following system of ODEs with space-dependent coefficients:

$$\frac{d}{dt}V(x,t)=-d_{ve}V(x,t)+b_{ve}+v_{ve}\frac{A_{1}^{2}(x)}{p_{1}^{2}+A_{1}^{2}(x)}$$ Eq 1

$$\frac{d}{dt}I(x,t)=-d_{in}I(x,t)+b_{in}+v_{in}\frac{A_{2}^{2}(x)}{p_{2}^{2}+A_{2}^{2}(x)}\frac{p_{3}^{2}}{p_{3}^{2}+V^{2}(x,t)}\frac{p_{4}^{2}}{p_{4}^{2}+D^{2}(x,t)}\frac{A_{1}^{2}(x)}{p_{5}^{2}+A_{1}^{2}(x,t)}$$ Eq 2

$$\frac{d}{dt}D(x,t)=-d_{do}D(x,t)+b_{do}+v_{do}\frac{p_{6}^{2}}{p_{6}^{2}+I^{2}(x,t)}\frac{p_{7}^{2}}{p_{7}^{2}+A_{2}^{2}(x,t)},$$ Eq 3

where *A*_*1*_ and *A*_*2*_ model the morphogen concentrations of Bmp and Edn1, respectively. All proteins are produced at a basal production rate *b* and a maximum production rate *v*, and are degraded at rate *d* (S2 Table). Gene regulation involves either up- or down regulating *v*, modeled by a Hill-function term with Hill-coefficient 2.

There are two ways to model the temporal order of patterning:

1. Vary the absolute values of *v* and *d* while keeping their ratio constant such that they reach the same steady state. A higher absolute value means a more rapid onset of gene expression that reaches the steady state more rapidly. This model assumes that temporal regulation is inherent to the minimal GRN, meaning that some genes respond quickly to signals while others are slower.
2. Include “if” statements in the GRN, such that certain genes are only expressed after a given time. This assumes that the temporal order is regulated by other mechanisms not included in the GRN.

In all experiments (except S12–S15 Figs) we use model 1 above, where we simulate the differences in the timing of V, I and D gene expression by varying production and degradation parameters, while keeping the ratio between production and degradation constant for all gene groups. Hence we use:

$$v_{in}>v_{ve}>v_{do}\;\mathrm{and}\;\frac{v_{ve}}{d_{ve}}=\frac{v_{in}}{d_{in}}=\frac{v_{do}}{d_{do}}=1$$ Eq 4

for the observed IVD pattern. See S2 Table for the parameter values of all temporal orders. The *d* and *v* values for the IVD pattern are chosen such that the onset of gene expression agrees with that in vivo.

For a 1D model of the arch without domain growth along the line of morphogen decay (i.e. the D-V axis), we discretize the domain from 0–70 nm equidistantly into *N = 201* nodes. Each node is exposed to individual morphogen concentrations depending on its position along the D-V axis and we then solve the GRN (Eqs 1–3) for each node, resulting in a system with 603 ODEs, which we solve numerically, using the Euler forward method with *Δt = 93*.*6 sec*. Simulation time *t = 0* corresponds to 22 hours postfertilization (hpf) of zebrafish development [46]. Since we are interested in temporal dynamics rather than a steady-state solution we do not run the simulations until steady state is achieved, but stop them at 35 hpf and plot the results.

We define a cell as “patterned” by a certain gene if its expression level is higher than 20% of the maximum and higher than all other genes S1 Movie.

#### The two-dimensional (2D) model

To extend the basic 1D model to include distinct cells, we represent the mandibular arch along both its D-V and anterior-posterior (A-P) axes. In this 2D model we represent cell centers by vertices $x_{i}\in R^{2},i=1,\ldots N$ (black dots in inset in Fig 1B). The Voronoi tessellation between the nodes represents the locations of cell membranes [55]. A generalized Morse potential between cell centers (white arrow in inset) accounts for cell-cell adhesion (attractive potential) and volume exclusion (repulsive potential)

$$V(\left\|x-x_{j}\right\|)=U_{0}e^{-\|x-x_{j}\|/\xi_{1}}-V_{0}e^{-\|x-x_{j}\|/\xi_{2}}.$$ Eq 5

This potential results in an equilibrium distance between cell centers

$$r_{0}=log(\xi_{2}/\xi_{1}*U/V)\;{(\xi }_{1}\xi_{2})/{(\xi }_{1}{-\xi }_{2}).$$ Eq 6

The attractive term in the potential guarantees the collective motion of cells, which is further directed by the deformation of the surrounding tissue, pushing cells in the direction of arch growth, and a global chemoattractant pulling the cells in the ventral direction. In our model, collective cell migration does not mean that cells are free to invade a previously empty space, but rather that the positions of the boundaries that keep the cells in proximity evolve, such that the whole structure migrates further away from the dorsal side of the embryo. To achieve an equal distribution of cells throughout the arch we include a global chemoattractant directing cell motion towards the ventral end in the model. While there have been many studies of the complex and heterogeneous mechanisms directing collective cell migration [56,57] e.g. the leader-follower model [58] responsible for the migration of cranial NC cells, the global chemoattractant together with deforming domain boundaries are sufficient to reproduce the observed tissue deformation and cell migration. For the time points 22, 24, 28, 32, and 35 hpf we measured actual arch boundaries from 6 different images and averaged these to compute a typical arch outline. Between these time points we evolve the arch outline by linear interpolation of the averaged boundaries. We define two rings of 2118 boundary nodes *b*_*i*_, which exert a repulsive force on the cell centers to keep the cells inside the arch (S1A Fig). At each time step we advance the nodes by the following ODE:

$$\frac{d}{dt}x_{i}=\nabla_{x}\sum_{j\neq i}V(\left\|x_{i}-x_{j}\right\|)+\nabla_{x}\left[\begin{array}{c} W_{1}\\ W_{2} \end{array}\right]\left[\begin{array}{c} e^{-\xi_{3}\|x_{i,1}-s_{1}\|}\\ e^{-\xi_{4}\|x_{i,2}-s_{2}\|} \end{array}\right]+\nabla_{x}\sum_{b_{i}}{Se}^{-\left\|x-b_{i}\right\|/\xi_{5}},$$ Eq 7

where the first term represents cell-cell interactions, the second term the chemoattractant, and the third term the force exerted by the surrounding tissue (see S3 Table for parameter values). At each time step the production zone of the morphogens is updated according to the current position of the surrounding tissue (green area in Fig 1B) and since the morphogens diffuse much faster than the cells move, we compute the quasi steady state of the morphogen gradients on a discrete 50x50 grid (white dots in Fig 1B), where, similar to the 1D model, we assume a constant production of morphogen and constant degradation across the whole domain (for an explicit visualization of the morphogen gradients, see S2 Movie). Each cell then experiences the morphogen gradient of the grid point closest to its center (Fig 1B green ring in inset) and this value acts as the input for *A*_*1*_*(x)* and *A*_*2*_*(x)* in the ODE system modeling the GRN, which we solve individually for each cell. Initially we place *N = 75* cells randomly inside the boundaries at 22 hpf and then move them once according to Eq 7 to equilibrate forces. This means that a simulation time of *t = 0* corresponds to a real time of 22 hpf. Then we solve the ODEs simulating the GRN (Eqs 1–3) for each cell and the motion equation (Eq 7) with the Euler forward method with Δt = 180 sec. Based on our imaging data cell number roughly doubles from 75 to 150 cells between 22 hpf to 35 hpf, most of which is due to cell divisions in the arch (~90%), with the remainder from NC cells continuing to migrate into the arch. To model cell division we randomly choose a cell that divides into two daughter cells at constant time intervals. Each daughter inherits the gene expression of the mother and we randomly place the new cell at a distance r_0_/20 from the mother cell. To account for continued NC migration into the arch in our simulation window (22–35 hpf) we add migrating cells to the dorsal end of the simulation domain at random locations along the dorsal domain boundary (white arrows in Fig 1B). Since these cells enter the arch dorsally we allow them to express dorsal genes at 40% of maximal. The time intervals of division and migration are computed such that the cell number doubles until 35 hpf in accordance with our measurements S3 Movie.

#### Boundary error and sensitivity

To quantify the error in the boundary position as a result of noise we average the domain boundaries obtained from three images each of *hand2* and *dlx5a* expression and compute the distance to cells at the boundaries in the model simulations. For each cell located at the boundary this gives one non-directional distance *d*_*i*_ (S1B and S1C Fig), and we sum over all cells located at the boundary to obtain a measure of the error of boundary positioning

$$E≔\sum_{i}d_{i}.$$ Eq 8

This error definition accounts for both large distances between the simulation boundary and the measured boundary, and for a long, ragged domain boundary opposed to the sharp boundary observed in live imaging (S2 Fig).

The sensitivity *si(t)* with respect to the parameters *p*_*i*_, *i = 1…7*, is defined as the derivative of each gene *y(t)* (representing *V(t)*, *I(t)*, and *D(t)*) with respect to p_i_

$$s_{i}(t)=\frac{\partial }{\partial p_{i}}y(t)$$ Eq 9

and fulfills the following ODE

$$\frac{\partial }{\partial t}s_{i}(t)=\frac{\partial }{\partial y}fs_{i}(t)+\frac{\partial }{\partial p_{i}}f.$$ Eq 10

We used the SUNDIALS suite of nonlinear differential equation solvers, with adaptive time stepping and integrated error control, to solve the ODEs of the GRN and additional ODEs for sensitivity [59].

#### Noise

We model noise in Bmp and Edn1 using Gaussian noise scaled with the morphogen concentration, such that

$$A_{i}(x)=max(0,A_{i}(x)+\eta_{i}\xi A_{i}(x))$$ Eq 11

where ξ∼N(0,1) and *i* = 1,2 and *η*_*i*_ is the strength of noise, with *η*_*1*_ = *η*_*2*_ = 1. Gaussian noise is also added to the ODEs, where *ν* = 0.05 models the strength of noise in gene regulation

$$\frac{d}{dt}V(x,t)=-d_{ve}V(x,t)+b_{ve}+v_{ve}\frac{A_{1}^{2}(x)}{p_{1}^{2}+A_{1}^{2}(x)}+\nu \frac{d}{dt}W$$ Eq 12

$$\frac{d}{dt}I(x,t)=-d_{in}I(x,t)+b_{in}+v_{in}\frac{A_{2}^{2}(x)}{p_{2}^{2}+A_{2}^{2}(x)}\frac{p_{3}^{2}}{p_{3}^{2}+V^{2}(x,t)}\frac{A_{1}^{2}(x)}{p_{5}^{2}+A_{1}^{2}(x)}\frac{p_{4}^{2}}{p_{4}^{2}+D^{2}(x,t)}+\nu \frac{d}{dt}W$$ Eq 13

$$\frac{d}{dt}D(x,t)=-d_{do}D(x,t)+b_{do}+v_{do}\frac{p_{6}^{2}}{p_{6}^{2}+I^{2}(x,t)}\frac{p_{7}^{2}}{p_{7}^{2}+A_{2}^{2}(x)}+\nu \frac{d}{dt}W.$$ Eq 14

We solve these with the Euler-Maruyama method and the same *Δt* as in the deterministic case.

## Results

### Intermediate (I) domain gene expression precedes ventral (V) and dorsal (D)

**Fig 2:**
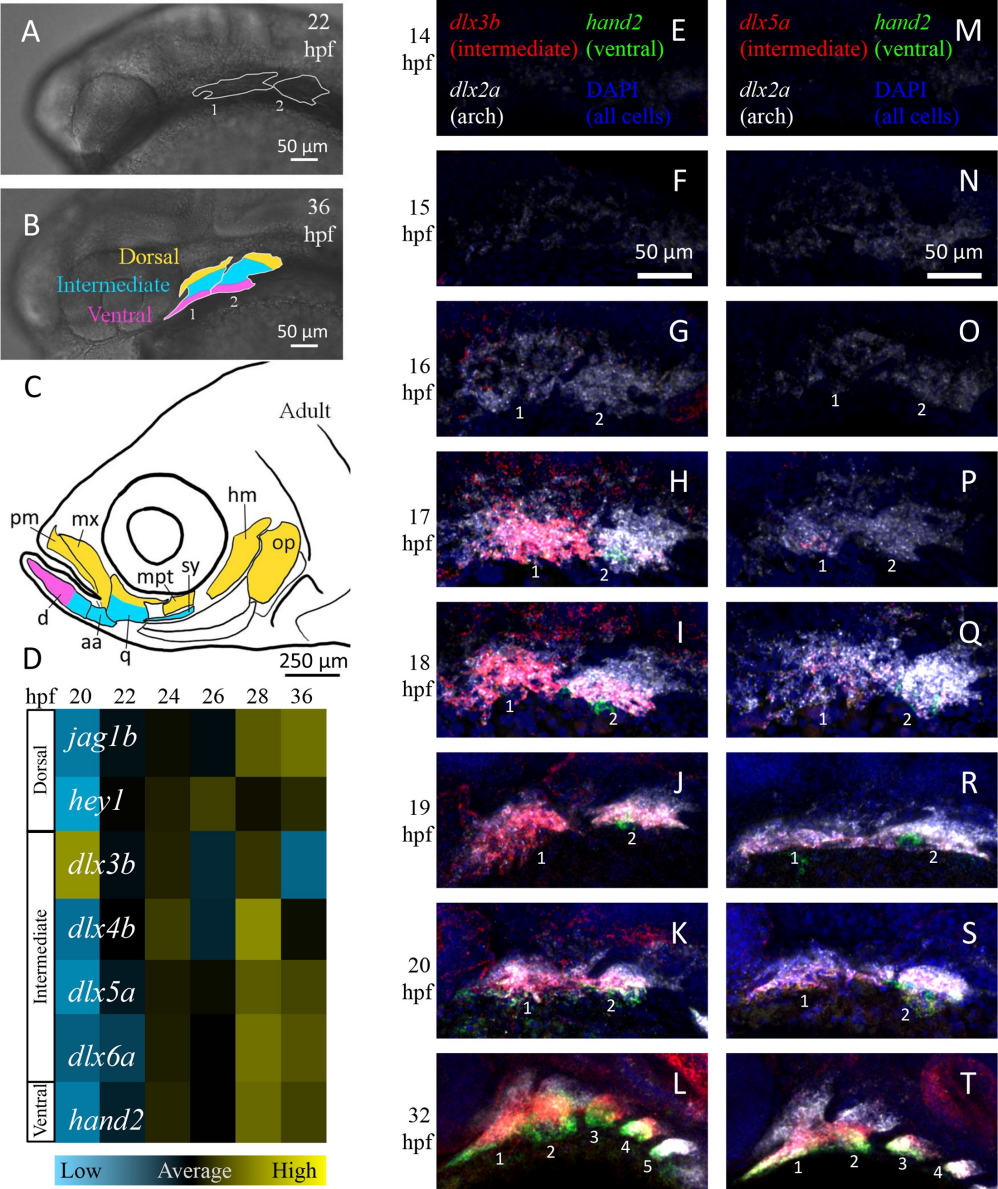
Expression of D-V arch patterning genes. (A-C) Morphogenesis of arches 1 (mandibular) and 2 (hyoid) in the zebrafish embryo. Lateral brightfield views of live embryos. By 36 hours postfertilization (hpf) the arch is patterned into distinct ventral (pink), intermediate (cyan), and dorsal (yellow) domains. These later give rise to lower jaw, joint and upper jaw elements, respectively. aa: anguloarticular, d: dentary, hm: hyomandibular, iop: interopercle, mx: maxilla, op: opercle, pm: premaxilla, pop: preopercle, sop: subopercle, sy: symplectic. (D) NanoString measurements of D-V patterning gene expression in zebrafish arches, normalized to per-cell levels and displayed as heat maps. n ≥ 3 biological replicates/time point. Expression of the intermediate factor *dlx3b* is highest at 20 hpf before other factors. (E-T) HCR *in situ* gene expression analysis. DAPI (blue) marks nuclei and *dlx2a* (white) marks neural crest. *dlx3b* (left column) and *dlx5a* (right column) expression are in red, and the ventral domain gene *hand2* is in green. *dlx3b* expression is first detected strongly at 17 hpf, while *dlx5a* expression appears at 18 hpf. *hand2* expression in the first arch is first detected at 20 hpf.

Neural crest (NC)-derived ectomesenchymal cells in pharyngeal arches 1 (mandibular) and 2 (hyoid) in zebrafish are patterned into three D-V domains between 14–36 hpf, which give rise to distinct skeletal elements in the adult (Fig 2A–2C). Arch D-V length roughly doubles over this period (from 30 to 60 μm). Previous in situ hybridization (ISH) studies have shown that *dlx3/4/5*/*6* are expressed together in an early ventral-intermediate (V-I) domain that later separates into V and I [24,29,30,42]. *hand2*, the homolog of which is induced by Dlx5/6 in mice [34,60,61], marks the new V domain and represses Dlx genes ventrally, restricting their expression to the I domain [42,62]. However, the precise timing of gene expression between 14–20 hpf, when these domains first appear, remains unclear.

To address this, we have measured transcript levels of seven D-V patterning genes in FAC-sorted arch cells using NanoString analysis (dorsal: *jag1b*, *hey1*; intermediate: *dlx3b*, *dlx4b*, *dlx5a*, *dlx6a*; ventral: *hand2*) (Fig 2D). Surprisingly, expression of *dlx3b* peaks early at 20 hpf, followed six hours later by three other intermediate genes (*dlx4b*, *dlx5a*, *dlx6a*), the ventral gene (*hand2*), and the dorsal gene *jag1b* at 26 hpf. Similarly with hybridization chain reaction (HCR) *in situs*, to facilitate co-localization and quantitation of expression (Fig 2E–2T) we find that within the domain of *dlx2a* expression, which marks NC cells in the entire arch, strong *dlx3b* expression is detected at ~17 hpf, at least an hour before *dlx5a* expression appears faintly at ~18 hpf. Meanwhile, *hand2* expression is not detected until ~20 hpf. Interestingly expression of *hand2* arises abruptly, while other genes such as *dlx5a* appear more slowly, yet both peak at a similar time point in the NanoString analysis. Thus, intermediate genes are the first to be expressed in the D-V sequence of arch patterning followed by ventral and finally dorsal genes.

### The computational model for the mandibular arch reproduces patterning observed in vivo

**Fig 3:**
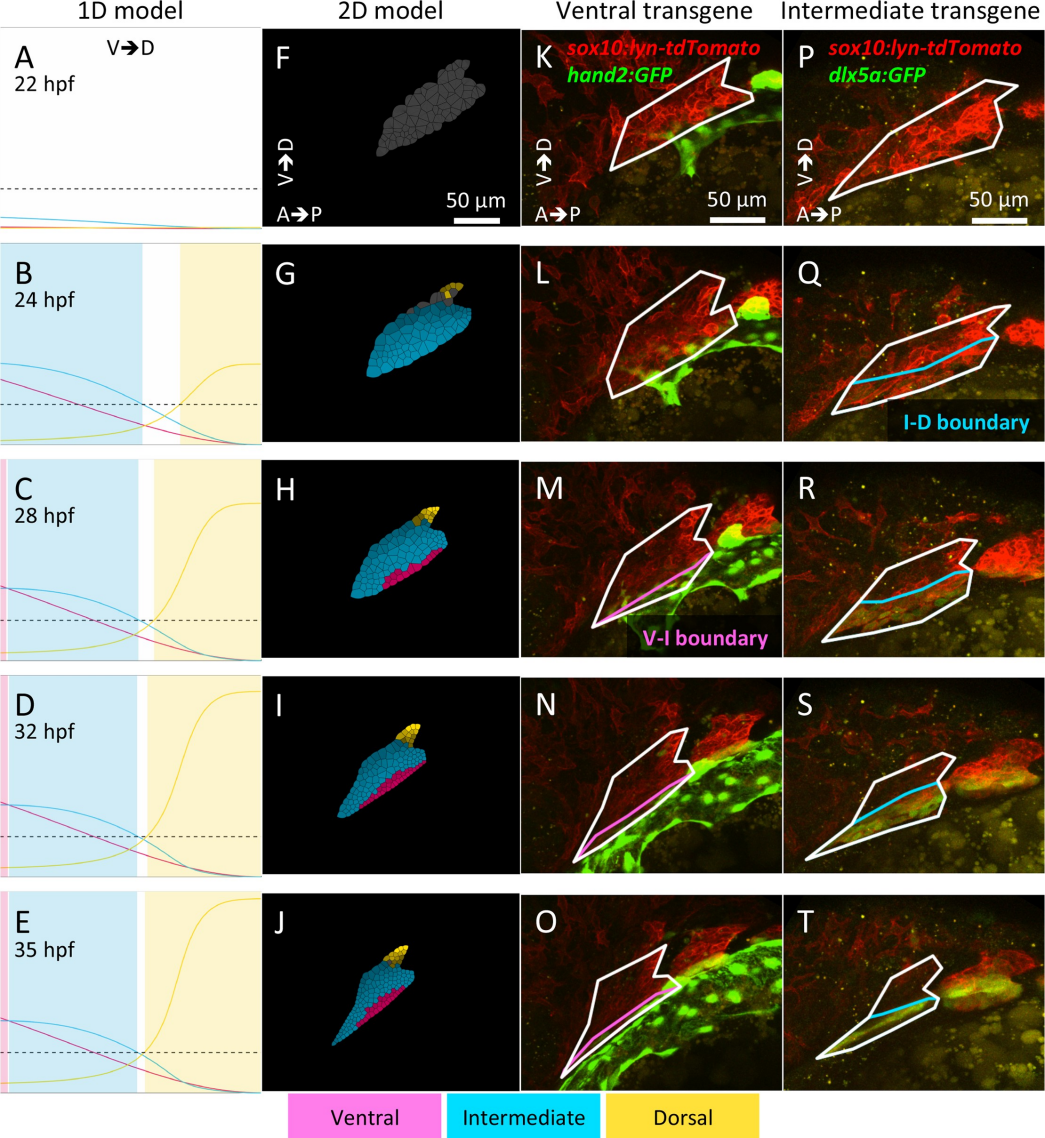
Both one- (1D) and two-dimensional (2D) models reproduce the spatio-temporal patterning observed *in vivo*. (A-E) Simulations from the 1D model of the mandibular arch along the D-V axis. A region is defined as patterned by a certain gene group if gene expression is above 20% of the maximum achieved in time (indicated by the black dashed line). White regions are not yet expressing any genes. The ventral domain (pink) remains narrow in the 1D simulation. (F-J) Simulations from the 2D model combining D-V and anterior-posterior (A-P) axes. Here a single cell is defined as patterned (indicated by a color) if the gene expression level exceeds 20% of the maximum, and grey indicates cells that are not yet expressing any genes above the 20% cut-off. Colors become deeper gradually as the level of expression increases. For animations of the model simulations, see S1, S2 and S3 Movies. (K-T) Images of transgenic embryos expressing the ventral marker gene *hand2*:*GFP* (K-O, S4 Movie) and the intermediate marker gene *dlx5a*:*GFP* (P-T, S5 Movie).

The 1D model (Fig 3A–3E) recapitulates the relative timing and sizes of D-V domains in the mandibular arch. Initially intermediate gene expression extends from the ventral end to approximately halfway up the D-V axis. Subsequently, dorsal genes are expressed at the dorsal end of the arch, leaving a section of “unpatterned” cells (white regions, in which gene expression is below the arbitrary 20% threshold) between I and D domains, which gradually diminishes. Initiation of ventral gene expression at 28 hpf creates a narrow V domain, which moves the I domain dorsally. The 2D model (Fig 3F–3J) captures these spatiotemporal dynamics of D-V domain formation. Here, individual cells are also defined as patterned if their gene expression exceeds 20%, and they are gradually colored correspondingly, such that grey indicates cells not yet expressing genes above the cut-off and more deeply colored cells indicate higher and higher levels of gene expression. Initially none of the cells express any of the genes above the 20% cut-off (grey cells). Between 22–35 hpf the arch elongates anteriorly and ventrally. During this tissue deformation the cells acquire D, I and V fates, (yellow, blue and pink, respectively) and form domains of the correct size and shape. The simulation results agree with live imaging of *hand2*:*GFP*:*sox10*:*lyn-tdTomato* double transgenics (Fig 3K–3O) or *dlx5a*:*GFP;sox10*:*lyn-tdTomato* double transgenics (Fig 3P–3T). We note that the boundaries of gene expression as shown by transgene reporter intensity are sharp (S2 Fig).

To generate a 2D cell-center based model that reflects arch morphogenesis as accurately as possible we have measured mandibular arch deformation in images of 6 *sox10*:*lyn-tdTomato* transgenic zebrafish embryos, the average of which is used to generate an arch outline (Fig 3K–3T). By further analyzing time-lapsed images of *sox10:nEOS* transgenics we find that: 1) cell number roughly doubles between 22–36 hpf, 2) ~90% of this increase in cell number is due to cell division and 3) only ~10% is due to cells migrating into the arch dorsally. These parameters are incorporated into the model to compute the time intervals of cell division and cell migration.

**Fig 4:**
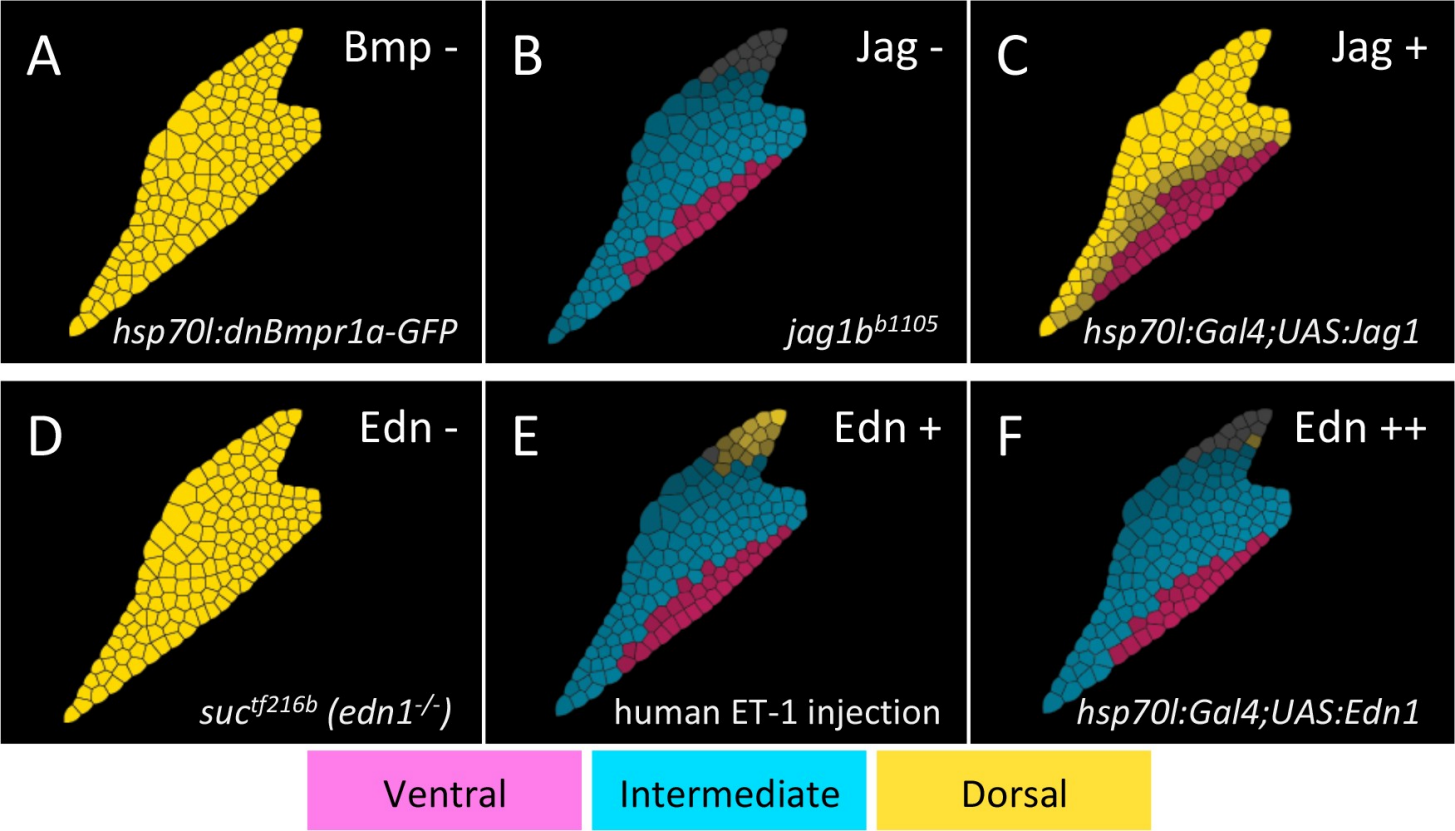
The 2D model reproduces experimental genetic perturbations. The modeling perturbation is shown at the upper-right and the matching genetic perturbation from the literature on the lower-right (normal patterning in Fig 3J). (A) Loss of Bmp signaling leads to a failure to express ventral (V, pink) and intermediate (I, cyan) and expansion of dorsal (D, yellow) genes. (B) Loss of Jag/Notch signaling leads to absence of the D domain. (C) Excess Jag/Notch signaling causes the D domain to expand at the expense of I. (D) Loss of Edn1 eliminates V and I, such that D expands. (E) Injecting recombinant human EDN1 into an *edn1*^*-/-*^ mutant leads to normal patterning. (F) Over-expression of Edn1 (5x) leads to loss of the D domain.

The 2D model also reproduces previously reported phenotypes of genetic or pharmacological perturbations that disrupt D-V patterning (Fig 4). V/I domains do not form and the D domain expands in embryos lacking Bmp or Edn1 signaling (reduced in the modeling simulations to 1% of wild-type expression [24,27] (Fig 4A and 4D). The I domain does not form and D expands ventrally in embryos overexpressing the dorsal factor Jag1 (500% of wild-type production) (Fig 4C). Conversely, the I domain expands to replace D in a *jag1-/-* mutant (1% of wild-type expression) or when Edn1 is overexpressed (500% of wild-type gradient maximum) (Fig 4B and 4F). Importantly, the model also recapitulates the normal D-V patterning observed experimentally with moderate, uniform Edn1 expression (50% of wild-type gradient maximum), achieved with Edn1 protein injections [24,27] (Fig 4E). Thus, despite the minimal GRN on which it was based, the model captures many aspects of patterning observed experimentally in vivo.

### Arch D-V patterning is sensitive both to the temporal order of domain formation and variation in Bmp signaling

**Fig 5:**
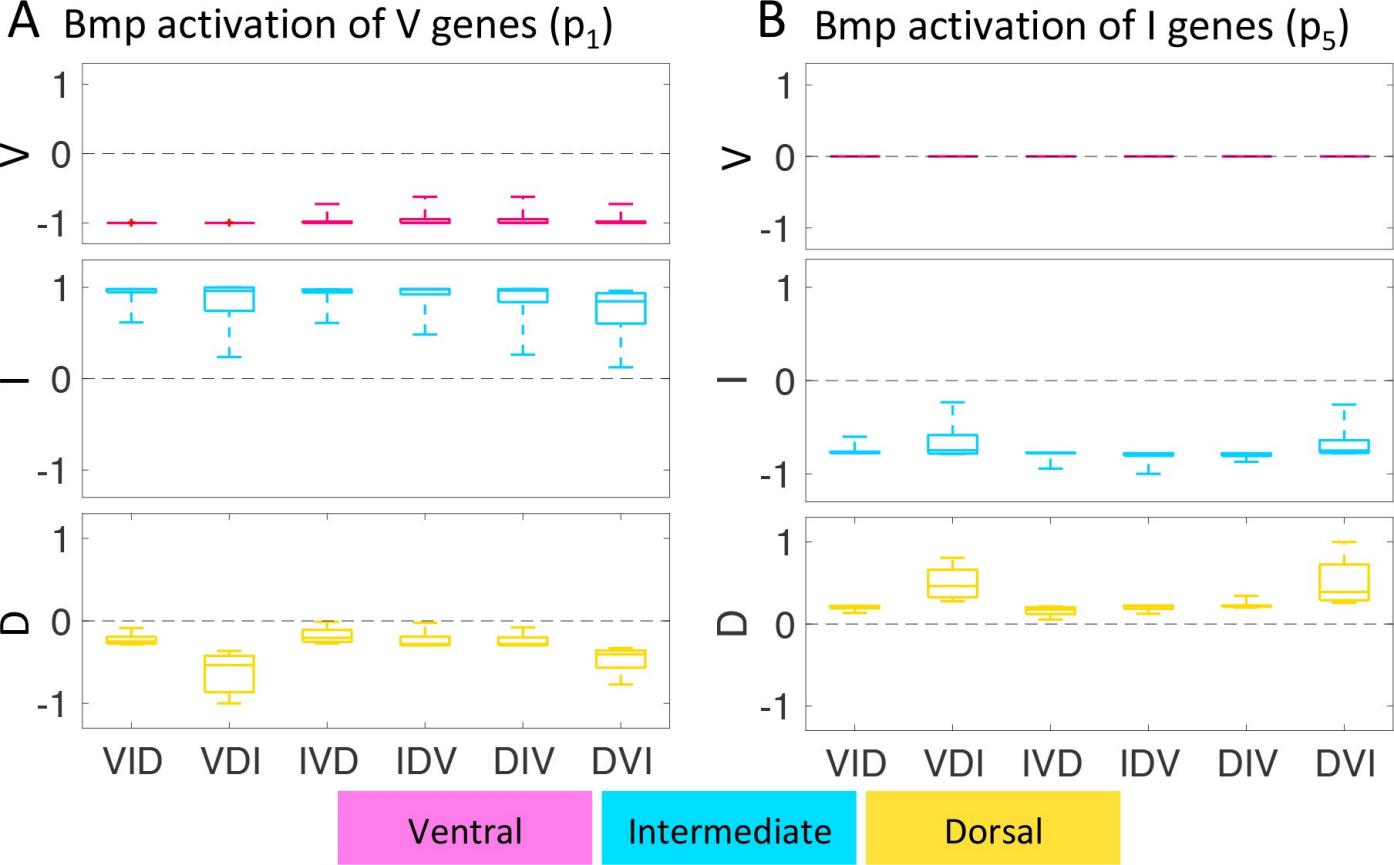
Sensitivity of gene expression with respect to perturbations in GRN parameters. Box plots summarize the sensitivities (*s*_*i*_*(t)*, Eq 9, y-axis) at 10 equidistant time points between 22 and 35 hpf and are normalized to the maximum overall sensitivities, for the ventral (V, pink), intermediate (I, cyan), and dorsal (D, yellow) genes for different temporal orders of expression (x-axis). The sensitivity value indicates how much the patterning output changes, and in which direction (i.e. positively or negatively), with a change in the given modeling parameter. Perturbations in the parameters for gene expression are most sensitive to Bmp activation of ventral genes (*p*_*1*,_ panel A) and to Bmp activation of intermediate genes (*p*_*5*,_ panel B) (see Fig 1A). For the full time dependent sensitivity data see S7 Fig and for complete box plots of all parameters see S4–S6 Figs. The median’s deviation from zero (black dashed line) indicates how sensitive a given temporal order is to perturbations in the respective parameter. The dorsal genes’ sensitivity to *p*_*1*_ and *p*_*5*_ depends more on the temporal order than ventral or intermediate genes. Expressing intermediate genes last (VDI and DVI) leads to the strongest sensitivity of the dorsal genes, since the medians deviate further from zero.

For any three sets of D-V patterning factors, there are six possible different temporal orders of gene expression (VID, VDI, IVD, IDV, DIV, DVI). Our gene expression studies indicate that *dlx3b* and its associated I domain appear first, so we asked how this order fares in our model as compared with other possible orders. By varying the production and degradation parameters for each gene group we simulate all six temporal orders (S1 and S2 Tables) and examine if any one is more robust than another. Changing the temporal order does not affect the final pattern at 35 hpf (S3 Fig). To compare sensitivity between the three temporal orders of gene expression we compute *si(t)* (Eq 9), evaluated at 10 equidistant time points between 22–35 hpf. We summarize the 10 resulting data points in box plots (Fig 5, S4–S6 Figs), where the line in the middle denotes the median, the bottom and top edges of the box the 25th and 75th percentile, respectively, and the whiskers extend to the most extreme data points. We normalize the data with respect to the highest value of *s*_*i*_*(t)* for both parameters p_1_ and p_5_ at all time points (for unprocessed, time-dependent data see S7 Fig). The distance between the median line and zero (dashed black lines) indicates how sensitive gene expression is to perturbations in the control parameters p_1_-p_7_, and the size of the box and length of the whiskers indicate how much the sensitivity changes with time. In general, all model simulations are relatively robust to parameter variations, indicating that the results are not due to the specific choice of parameters.

When cells are exposed to morphogen concentrations typical for the V and I domains (high Bmp and Edn1 concentrations) the GRN is most sensitive to perturbations in the BMP signaling parameters *p*_*1*_ (activation of V genes) and *p*_*5*_ (activation of I genes) (Fig 5; S4 and S5 Figs), and less so to variations in Edn1 signaling parameters *p*_*2*_ and *p*_*7*_. This is particularly true in cases where I genes are expressed last (VDI and DVI), since median sensitivity levels of dorsal genes deviate further from zero (dashed black lines; Fig 5 and S4 and S5 Figs). However, when cells are exposed to low Bmp and Edn1 concentrations (S6 Fig), typical for the D domain, perturbations in the Bmp gradient only affect the ventral genes, while intermediate and dorsal genes are sensitive to perturbations in the Edn1 parameters *p*_*2*_ and *p*_*7*._ This agrees with published evidence that Edn1 plays a primarily permissive role in ventral and intermediate gene expression [27,40]. A permissive role for Edn1 is further evident when we compute the sensitivity to parameter variations in the GRN if only one of the two morphogen gradients is present (S8 Fig). The results are similar to those for two morphogens in all three V, I and D domains. In all cases, the most crucial parameter is the one controlling the effect of the morphogen on the I domain, *p*_*2*_.

### Noise in Bmp, Edn1 or the downstream GRN has distinct affects on patterning

**Fig 6:**
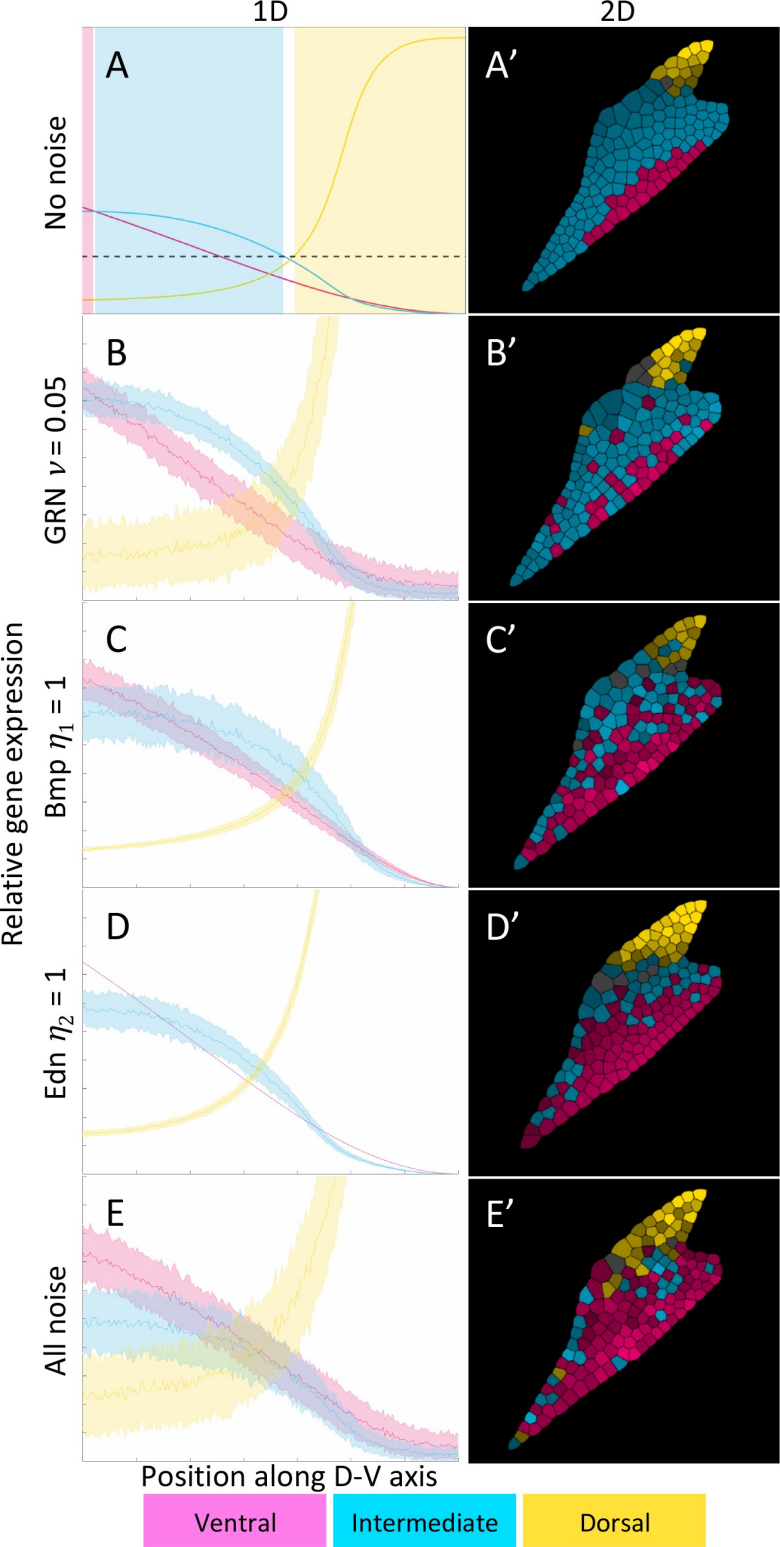
Different sources of noise have distinct effects on the gene expression profiles and domain patterning. Left column: 1D. Combined statistics from 100 simulations. The thick line is the mean value and the shaded area is ±σ. Right column: 2D. All panels show final states resulting from one simulation. (A-A’) No-noise reference (adapted from Fig 3E and 3J). (B-B’) Noise in the GRN disrupts boundaries of gene expression in all three domains, ventral (V, pink), intermediate (I, cyan), dorsal (D, yellow), while the mean value of the expression profile is preserved. (C-C’) Noise in Bmp signaling does not disrupt D and V slightly expands dorsally. (D-D’) Since Edn1 plays a permissive role noise in its gradient acts in one direction such that the I domain is partially replaced by V. (E-E’) When all three sources of noise are present simultaneously the effects are additive and the I domain is nearly lost while all three gene groups show strong fluctuations in their expression profiles as with noise only in the GRN. For animations see S6 and S7 Movies.

**Fig 7:**
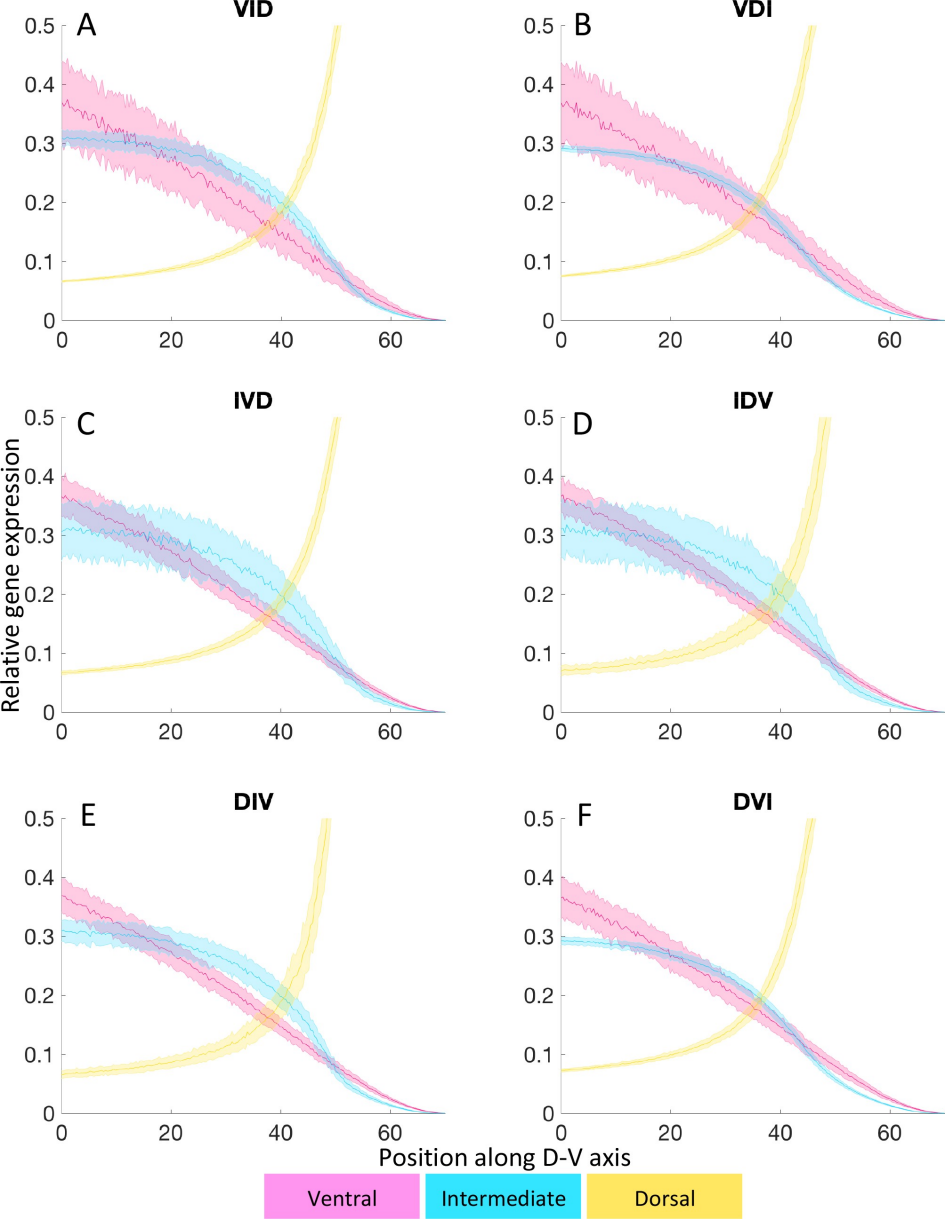
The temporal order of D-V domain formation modulates responses to noise in Bmp signaling. Each panel shows results of 100 simulations in the 1D model. The thick line is the mean value and the shaded area is ±σ. For the fully deterministic modeling result of IVD with no noise, see Fig 3E. (A,B) When the V domain is patterned first it is the most sensitive to noise in Bmp signaling. (C,D) Similarly, when the I domain is patterned first noise in Bmp affects the early I genes most strongly. (E,F) When the D domain is patterned first Bmp noise is more efficiently absorbed by the GRN leading to the most robust gene expression profiles.

To simulate the stochasticity that may occur in vivo, we have added Gaussian noise to the morphogen gradients and to the GRN (Eqs 11–14, Figs 6 and 7, S9–S11 Figs and S6 and S7 Movies). To compute a large number of stochastic simulations for statistics, and for better visualization, we investigate the effects of noise first in the 1D model. We plot the mean (thick lines) and ±σ (shaded regions) over 100 simulations (Fig 6B–6E, 6A for comparison without noise). For the 2D model, we show end-states for single simulations (Fig 6B’–6E’, 6A’ for comparison without noise). The simulations reveal that noise in Bmp signaling is transmitted differently than noise in Edn1 into the GRN. While intermediate gene expression is affected by both signals, ventral gene expression is not affected by noise in the Edn1 gradient, since the only input into the V domain is the Bmp gradient. Dorsal genes are most robust to morphogen fluctuations (Fig 6C–6D’), since they are mostly controlled indirectly through the ventral genes, but they are the most sensitive to gene regulation noise (Fig 6B and 6B’). Since Edn1 is required for intermediate gene expression, but increasing Edn1 signaling does not expand the I domain (Fig 4E and 4F), fluctuations in Edn1 only act in one direction, meaning that lower Edn1 levels due to noise reduce the I domain but higher levels have no effect. As a result the I domain is reduced with fluctuations in Edn1 (Fig 6D and 6D’), when compared to the deterministic case (Fig 6A and 6A’, S9 Fig) in both 1D and 2D models. This means that Edn1 acts “unidirectionally” as a permissive factor. Gene expression noise appears to be the strongest driver of fluctuations in patterning, since even relatively small fluctuations (*ν = 0*.*05*) in the GRN alter gene expression profiles substantially (Fig 6B and 6B’). The effects of individual fluctuations are additive when all sources of noise are combined (Fig 6E and 6E’, S11 Fig), i.e. both increased fluctuations in dorsal gene expression due to GRN noise, and expansion of the V domain due to noise in the Edn1 gradient.

### The precision of boundary positioning depends on the temporal order of D-V domain formation

Precision refers to the level of variation in boundary positions in stochastic simulations. Similar to our analysis of sensitivity to different parameters in the GRN, we examine if the sequence of D-V domain formation influences precision, as a measure of robustness in response to noise. When fluctuations are limited to the Bmp gradient the genes expressed first absorb most of the noise (Fig 7). The exception is when dorsal genes are expressed earliest, which are in general the most robust to Bmp noise, such that the DIV and DVI sequences are the least susceptible to Bmp fluctuations (Fig 7E and 7F).

This is in contrast to noise in the GRN (*v = 0*.*05*), where genes expressed earliest are the most robust and genes expressed later are more susceptible to noise (S9 Fig). This is particularly true when genes expressed in the I domain are first in the D-V sequence. This indicates that patterning I first is beneficial since fluctuations in intermediate gene expression affect both the precision of the V-I and I-D boundaries. With fluctuations in the Edn1 gradient, the I domain is severely reduced when intermediate genes are expressed last, while there is still a distinct I domain in the case of either V or I being expressed first (S10 Fig), further indicating that an early expression of intermediate genes is beneficial for boundary accuracy. Noise in the Edn1 gradient has the most similar effect across the 6 possible temporal orders. Early expression of intermediate genes, however, leads to slightly stronger fluctuations in their expression as a result of Edn1 noise, while still preserving a distinct I domain. Due to the different effects of the three sources of noise in the context of different temporal orders, combining all sources of noise indicates that a later onset of intermediate gene expression leads to the strongest fluctuations and higher sensitivity (S11 Fig).

When differences in the temporal order are enforced explicitly in the simulations (S12 Fig), the responses to the different sources of noise are similar to the case of intrinsic regulation, only with a more severe loss of the I domain due to noise in Edn1 (S13 Fig). However we do not see any distinction between the temporal orders in the extrinsic model with noise in the Bmp gradient (S14 Fig) in contrast to the intrinsic model with Bmp noise (Fig 7).

### The accuracy of boundary positioning depends on the temporal order of D-V domain formation

**Fig 8:**
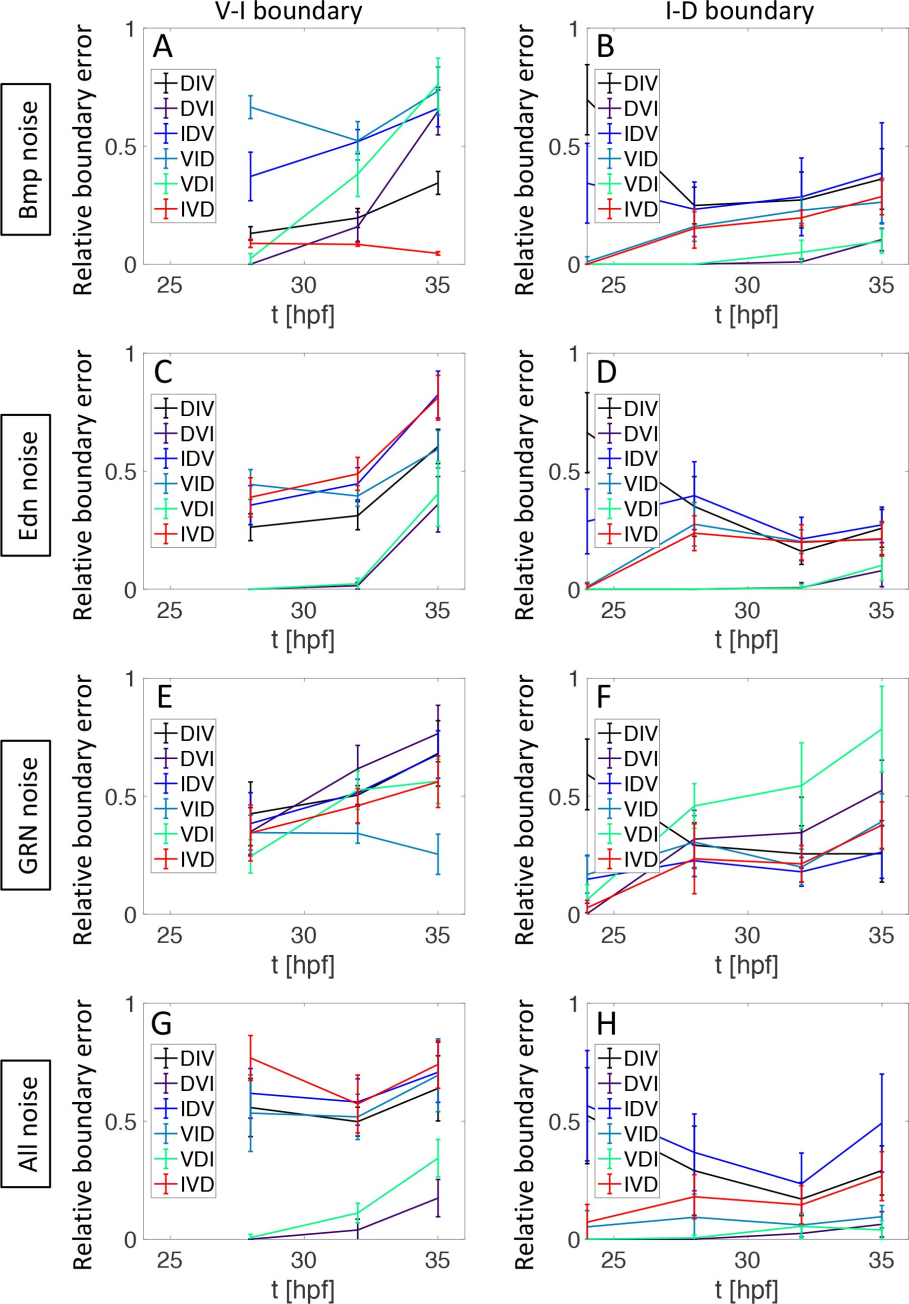
The temporal order of D-V domain formation modulates responses to noise and the accuracy of domain boundaries. Graphs show the boundary error *E* between simulation results and measurements in wild type. Statistics are collected for 10 simulations with the 2D model and results are normalized with respect to the highest error. The line shows the mean value and the error bars ±σ. (A,B) For noise in Bmp signaling the IVD order allows more robust positioning of the V-I boundary (A). Forming I last is however slightly advantageous for positioning the I-D boundary (B). (C,D) For noise in Edn1 signaling forming I last allows more robust positioning of both boundaries. (E,F) With noise in the GRN the boundary error still depends on the temporal sequence but there is no clear clustering with early or late gene groups. (G,H) With all three sources of noise combined it is clearly advantageous to express I genes last for the positioning of the V-I boundary (G) and slightly advantageous for the I-D boundary.

Accuracy refers to boundary positions relative to the measured wild-type positions (Fig 8). We simulate 10 runs of the 2D model, compute statistics of the boundary error *(E)* in Eq 8, normalize according to the highest value and plot the mean (line) and ±σ (error bars). Accuracy depends strongly on which gene group is expressed last, especially for the I-D boundary. When either Bmp or Edn1 are noisy, the I-D boundary is positioned most accurately when I is last, slightly less accurate when D is last and inaccurate when V is last (Fig 8B and 8D). In contrast, when Bmp is noisy the V-I boundary shows no clustering of temporal orders of patterning (Fig 8A), but when Edn1 is noisy accuracy increases when I is last (Fig 8C). The boundary accuracy depends less distinctly on the temporal order of patterning when the gene regulation is noisy (Fig 8E and 8F). When we combine all sources of noise a late appearance of the I domain clearly improves positioning of the V-I boundary and slightly improves the I-D boundary (Fig 8G and 8H).

In general the domain boundaries are more sensitive to fluctuations in Bmp than in Edn1, especially at the V-I boundary. Presumably the mutual inhibition between intermediate and dorsal genes makes the I-D boundary more robust to stochastic fluctuations. When the sequence of patterning is controlled by extrinsic factors not inherent in our minimal GRN (S12 Fig), the accuracy of boundary positioning is more sensitive to individual sources of noise in all cases except for positioning the V-I boundary with fluctuations in only Edn1 (S15 Fig). However, with the external control of gene expression timing the noise effects appear to be less additive and the extrinsic model positions the boundaries more accurately.

### Patterning involves a trade-off between precision and accuracy

**Fig 9:**
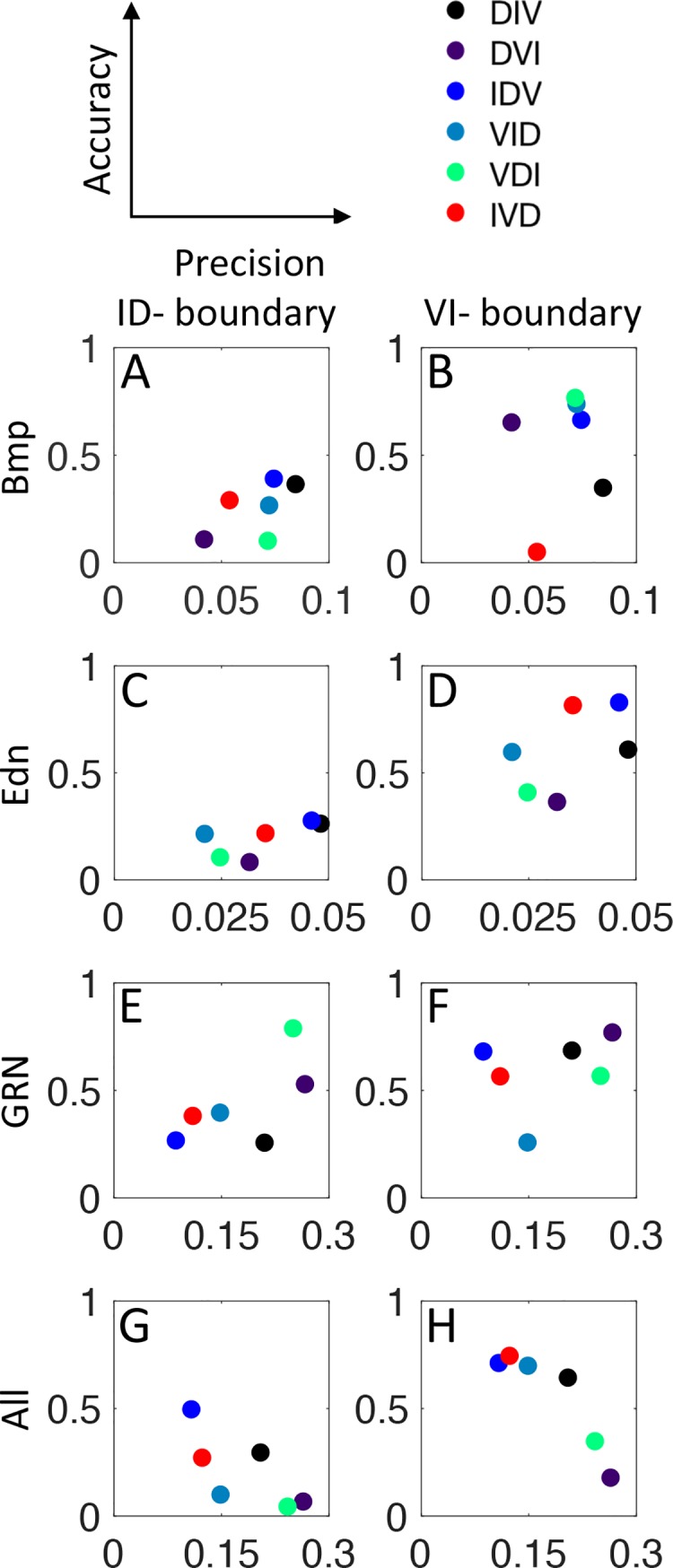
Temporal orders have different trade-offs for domain patterning in the context of noise. Each graph shows the precision in gene expression on the x-axis and the accuracy of simulated boundaries relative to measured wild-type boundaries on the y-axis. The contribution of noise is shown both individually for Bmp (A,B) and Edn1 (C,D) signaling and the GRN (E,F), and also for all sources of noise combined (G,H). For both precision and accuracy, a lower numerical value reflects better resistance to noise. For all noise sources combined (G,H) the data align on the anti-diagonal indicating trade-off between accuracy and precision. The observed IVD order (red dots) favors precision at the expense of accuracy.

From the above results, different temporal orders lead to different degrees of noise in gene expression profiles (Figs 6, 7 and S9–S11), while there are observed differences in accuracy of domain boundary positioning (Fig 8). We now quantify these effects to investigate how accuracy and precision relate to each other at both domain boundaries, and with the different sources of noise (Fig 9). Precision is measured as the maximal standard deviation (Figs 7 and S9–S11), corresponding to the maximal width of the shaded regions. Accuracy is the mean value of the boundary error (Fig 8). Hence for both, a low value indicates higher accuracy or higher precision. When all sources of noise are included in the simulation, an anti-diagonal trend is observed, indicating a trade-off between precision and accuracy, where temporal patterns with more precise boundary positioning have less accuracy, and vice versa. The observed IVD pattern favors precision over accuracy at both boundaries, suggesting that the patterning system has evolved to maximize precision in gene expression domain boundaries.

## Discussion

We have analyzed spatiotemporal patterns of expression of D-V patterning genes during pharyngeal arch morphogenesis in zebrafish embryos and combined our experimental observations with published data to generate the first computational model of the developing mandibular arch. Previous efforts have compiled information from imaging and gene expression databases, such as the FaceBase consortium [63], and analyzed cellular behavior during craniofacial morphogenesis [20], but ours is the first to integrate spatiotemporal gene expression patterns with morphogen gradients, tissue measurements, and known mutant phenotypes. Our model captures many of the spatial and temporal features of arch development and recapitulates genetic perturbations. It also provides novel insights into developmental constraints on the system, including: 1) Bmp is responsible for providing positional information to the cells, while Edn1 is permissive, 2) the temporal order of patterning is important for the system’s capacity to account for noise, and 3) the temporal order favors precision over accuracy in boundary positioning.

Many developmental processes that involve periodic patterning reflect underlying reaction-diffusion systems that deal efficiently with noise through their intrinsic feedback loops [12,16,19]. We find that the temporal order of gene expression provides a previously unappreciated factor in improving responses to noise. Surprisingly in our experiments it is the intermediate gene *dlx3b* that is the first detected at 16–17 hpf by HCR and slightly later (20 hpf) in our NanoString analyses, which may reflect the fact that sorted cells used for NanoString are derived from multiple arches (mandibular, hyoid, branchial) while with HCR we image the first arch directly. *dlx3b* might serve as an early response factor, possibly integrating input from multiple signals and priming other patterning genes in the GRN. The early appearance of the I domain means that the arch is not patterned consecutively from one end to the other (i.e. the VID or DIV orders), but rather in the peculiar sequence of establishing the middle first. Intuitively one can assume that patterning the central domain first already establishes both boundaries. However, that is not the case here either, since expression of the intermediate genes initially extends to the ventral end of the arch and is only later replaced by the ventral genes, such that both boundaries are established independently and non-simultaneously. We have investigated if this temporal order is beneficial for the response of the system to stochastic fluctuations. Our model simulations suggest that avoiding having intermediate genes expressed last improves the robustness to perturbations in Bmp signaling parameters and precision in the positioning of D-V domain boundaries. While *dlx3b* knockdown does not cause significant patterning defects, this could reflect compensation by *dlx4b* or *dlx5a* [42]. The unique function of *dlx3b* in the GRN is supported by its distinct spatial expression pattern from the *dlx5/6* pair and also from its neighboring *dlx4* cluster counterpart [42,64–66].

Our results suggest that altering the temporal order of D-V gene expression, (e.g. using optogenetic approaches to induce expression of *hand2* in its normal spatial domain but prior to onset of intermediate gene expression and thus creating a VID sequence) will disrupt the accuracy and robustness of D-V domain boundaries. Future studies are also needed to determine if the temporal order of gene expression in this system is controlled by gene-intrinsic differences in sensitivity to signals (as is the case in our minimal model), or if *trans*-acting factors play a greater role.

We also note that the precision and accuracy of D-V domain boundary formation are distinctly susceptible to noise, with the boundary between V and I domains especially sensitive to noise in Bmp signaling or in the downstream GRN. Precision and accuracy in the boundaries of gene expression domains are both goals for patterning systems, but our model reveals divergent paths to reach each of those goals in the mandibular arch. We find that the most precise gene expression profiles in response to Bmp signaling are achieved when dorsal genes are expressed first (Fig 7), while the most accurate boundaries were obtained when I genes were expressed last (Fig 8). Instead, our data in zebrafish indicate that I genes are expressed first, reflecting a trade-off that seems to favor precision over accuracy (Fig 9). It is important to note here that the model does not achieve high boundary positioning accuracy for the I-D boundary with any of the temporal patterns even in the absence of noise (S1A Fig). This might be a result of measurement inaccuracy and due to the projection of a 3D geometry into 2D, or that other factors not included in our minimal GRN are responsible for pushing the I-D boundary more towards the ventral end. Such trade-offs between precision and accuracy in other systems typically involve negative feedback [12,18,67], while it is temporal gene expression control that navigates this tradeoff in our model. Future analysis will reveal if feedback loops also operate at arch domain boundaries.

Our modeling results for precision and accuracy derive from combinations of 10–100 simulations, somewhat analogous to variability in patterning that can occur between individual embryos. We speculate that developmentally this suggests that patterning will favor precision (i.e. show a narrow range of variability between individuals), even if that range differs from the species ideal. Evolutionarily, this might permit rapid variation in craniofacial morphology during radiation events while still maintaining intra-population characteristics. Changes in craniofacial structures are one of the most striking adaptations in rapidly evolving vertebrate populations such as African rift lake cichlids, and it would be interesting to examine the parameters of patterning in these species [68,69].

Bmp and Edn1 signaling have overlapping but distinct functions in D-V patterning of the mandibular arch, with Edn1 primarily maintaining intermediate gene expression and playing a permissive rather than instructive role [24,27,30,40]. Our modeling results are consistent with Bmp signaling acting as the principal instructive ventralizing signal, at least for inducing V and I domains. They also suggest that moderate perturbations or noise in Bmp signaling, at levels that do not completely eliminate expression of various factors or formation of later cartilage structures, will still have effects on D-V arch patterning. In several reports, changes in levels of patterning signals result in a moderate-to-severe spectrum of phenotypes. Reduction in Bmp signaling by heat-shock induction of a dominant-negative Bmp receptor can lead to varying degrees of loss of ventral structures, depending on the timing of heat-shock and the dose of the dominant-negative transgene [24,30,70]. Increasing Bmp levels moderately by overexpression of Bmp ligand or injection/bead insertion of Bmp proteins causes homeotic transformations, such as the more dorsal palatoquadrate cartilage acquiring characteristics of the ventral Meckel’s cartilage, while more severe changes in signaling levels leads to significant losses throughout the jaw cartilage [24,30,71]. Modulation of Edn1 signaling in either direction similarly results in varying degrees of patterning changes, although consistent with its permissive role even very high levels of Edn1 do not expand the I domain, while reducing the D domain [24,26,27,30,39].

These divergent phenotypes, along with our analyses of parameter sensitivity and the effects of noise, suggest a region of stability where the patterning network can compensate for changes in signals, with moderate phenotypes appearing at the edges of this region, and severe phenotypes occurring when signaling falls outside the stable region altogether. Our computational model suggests that further experiments up- and down-regulating signaling will reveal transition points where stability breaks down, and those data will in turn improve the quality of our model. We can also extend our framework in the future to incorporate other signals that have been implicated in D-V patterning and growth, including Wnts [72] and Fgfs [73] as well as other GRN components [40,42].

Boundaries between different D-V domains in the mandibular arch are sharp, which is especially prominent in our live imaging (S2 Fig). How is this sharpness achieved? Our model simulates sharp domain boundaries in the absence of noise. However, when stochastic fluctuations are present the domain boundaries are no longer sharp since all cells are responding independently to the noisy signals. Computationally, we can extend our model to investigate if cell-cell communication and cohesion between cells of the same identity will improve boundary sharpening. Experimentally, we can examine if changes in signaling too small to disrupt overall patterning can nevertheless degrade boundary sharpness, which would suggest that this sharpness comes from the patterning pathways themselves. Although our current analysis of live cell dynamics does not suggest a significant degree of NC cell rearrangements once they have reached the pharyngeal arches, cell sorting contributes to domain boundary sharpening in some contexts, such as the neural tube [74], and automated tracking of large numbers of arch NC cells in the future might reveal subtle but important cell movements in arches as they are patterned.

D-V arch patterning of the mandibular arch and its associated GRN are largely conserved among vertebrates [21,23,75]. However, differences in the resulting anatomy of the jaw and skull in the adult, as well as the size, shape, and growth of the mandibular arch primordium make it difficult in some cases to draw clear homologies across species e.g. zebrafish and mouse or human [21,23]. In addition, differences in the timing of gene expression and our ability to identify clear functional homologues across species have made comparisons of the genetic pathways involved challenging. Having made a computational model for the zebrafish jaw that incorporates these spatiotemporal features we can now extrapolate to other species to determine if similar constraints apply. In addition, we can begin to ask questions at a more integrated, systems biology level, about how such changes in size, shape and timing may have arisen and how the GRN has adjusted to compensate for changes in such features as signal propagation and noise.

## Supporting information

S1 Table Microscopic dissociation parameters. The microscopic dissociation constants p_i_ used to generate the mandibular gene regulatory network (GRN) for dorsal-ventral (D-V) patterning and the literature references on which the interactions are based (see Fig 1A and Eqs 1–3).
(DOCX)

S2 Table Production and degradation parameters. Production/degradation rates for the ventral (ve) intermediate (in) and dorsal (do) genes. One value is given since the production rate equals the degradation rate for each gene group.
(DOCX)

S3 Table Mechanical parameters. Parameters used to model mechanical forces between cells, between cells and surrounding tissues, and chemoattraction.
(DOCX)

S1 Fig Enforcing the boundary conditions and modeling the domain boundary error. (A) Simulation results of the two-dimensional (2D) model with the measured boundary (green line), averaged over 6 sets of zebrafish images. To enforce the simulated cells to stay inside the measured arch outlines (green line) we put two rings of a total of 2118 boundary nodes around the outline (white lines). These nodes exert a repulsive force (Eq 7) on the cell centers x_i_, such that the cells do not leave the green perimeter. (B) Simulation results of the 2D model overlayed with the domain boundaries averaged over three sets of images from zebrafish embryos for the V-I (pink-white dashed line) and I-D (yellow-white dashed line) boundaries. (C) Boundary error E is the sum of the distances di (green arrows) between the simulated domain boundary for each cell at the boundary (white dots) and the actual measured boundary (white line).
(TIF)

S2 Fig Domain transgene expression boundaries are sharp. Single confocal z-slices of *dlx5*:*GFP*;*sox10*:*lyn-tdTomato* (A) and *hand2*:*GFP*;*sox10*:*lyn-tdTomato* (B) double-transgenic embryos at 30 hpf. For both the intermediate-dorsal boundary (A) and ventral-intermediate boundary (B), quantification of per-cell fluorescence intensity in arches 1 and 2 reveals two distinct populations of cells, those with high signal intensity and those with low signal intensity, indicating an abrupt drop-off in fluorescence signal and thus a sharp boundary of transgene expression. In the graphs, the *y*-axis shows mean GFP intensity per cell, normalized to the maximum possible intensity. The *x*-axis shows the D-V position of each cell, measured in μm with the ventral edge of the arch at 0.
(TIF)

S3 Fig 2D modeling results for six different temporal orders of D-V domain formation. By simply switching the absolute values of the production/degradation rates while keeping their ratio constant (see S1 Table), the three gene groups (dorsal, intermediate and ventral) can be expressed in any of the 6 possible orders and still lead to a correct final pattern at 35 hpf.
(TIF)

S4 Fig Model parameter sensitivities over time at high morphogen concentrations. Sensitivity plots for all gene groups with respect to the parameters p1−p7 (see Fig 1A), when cells experience morphogen concentrations typical of the V domain. The values are normalized with respect to the strongest sensitivity over time in all of the parameters for each gene V,I and D individually. The Bmp parameters p1 and p5 are most sensitive to fluctuations (see Fig 5). The temporal order mostly effects the sensitivity of the dorsal domain, where expressing I last (center) leads to the strongest sensitivity of D.
(TIF)

S5 Fig Model parameter sensitivities over time at intermediate morphogen concentrations. Sensitivity plots for all gene groups with respect to the parameters p1 − p7 when cells experience morphogen concentrations typical of those in the I domain. The values are normalized with respect to the strongest sensitivity over time in all of the parameters for each gene V, I and D individually. The GRN is again most sensitive to perturbations in the parameters modeling the BMP effect (p1 and p5), though the sensitivity of I and D with respect to p1 has been reduced compared to the ventral domain, due to the lower concentration of Bmp. The temporal order is again mostly significant in the dorsal domain, where expressing I last (center) leads to the strongest sensitivity of D.
(TIF)

S6 Fig Model parameter sensitivities over time at low morphogen concentrations. Sensitivity plots for all gene groups with respect to the parameters p_1_ − p_7_, when cells experience morphogen concentrations typical to those in the D domain. The values are normalized with respect to the strongest sensitivity over time in all of the parameters for each gene V, I and D individually. Since both the Bmp and ventral gene concentrations are very low in the dorsal domain, the intermediate gene expression is here most sensitive to the direct control by the two morphogens (Edn1 (p_2_) and Bmp (p_5_), see Fig 1A). Similarly the dorsal gene expression depends on those two parameters by indirect interaction through the intermediate genes. The temporal order here favors a late expression of intermediate genes (center).
(TIF)

S7 Fig Certain model parameters are more sensitive to the temporal order of D-V domain formation over time. The normalized sensitivities *s*_*i*_*(t)* plotted over time (*x*-axis) for genes expressed in the D, I and V domains with respect to the parameters p_1_ (left plots) and p_5_ (right plots). These parameters both model the influences of BMP (see Fig 1A) and are the most sensitive to the domain order. The sensitivity to both parameters p_1_ and p_5_ deviates the most from zero at early times, when genes in the I domain are expressed last (VDI and DVI).
(TIF)

S8 Fig Modeling a single morphogen system shows that it is robust. (A) The GRN used to compute gene expression inside each cell if only one D-V morphogen gradient emanating from ventral is controlling the patterning. The parameters p2 and p5 in Fig 1A merge into only p2 here. (B,C) The control parameters pi when the single morphogen gradients is either Bmp (short range) or Edn1 (long range). (D) The sensitivities in a two morphogen GRN for comparison with the IVD order and morphogen concentrations typical for the ventral domain (see also S4 Fig, IVD). (E, F) The sensitivity of the three gene groups when only one gradient with extent similar to Bmp or Edn1, respectively controls the GRN. The parameter p1 is still the only one with effect on ventral gene expression and p2 now accumulates the sensitivity of the intermediate and dorsal genes compared to p2 and p5 in the two morphogen model.
(TIF)

S9 Fig Early-expressed genes are more robust. 1D gene expression profiles for ventral (pink), intermediate (blue) and dorsal (yellow) genes showing that with noise in the GRN, ν = 0.05. The thick lines show the mean value over 100 simulations and the shaded area is ±σ. Genes expressed earliest are more robust and less susceptible to noise than genes expressed later. Especially in the case when I is expressed last (center) the GRN is most susceptible to noise, in agreement with the findings in S4 Fig and S5 Fig.
(TIF)

S10 Fig Effects of noise in the Edn1 gradient are similar with different temporal orders of domain formation. 1D gene expression profiles for ventral (pink), intermediate (blue) and dorsal (yellow) genes with noise in Edn1, η2 = 1. The thick lines show the mean value over 100 simulations and the shaded area is ±σ. The simulations show that Edn1 fluctuations affect the intermediate gene group most strongly, especially when I is expressed first (right). Although Edn1 noise has a smaller effect on the deviation in expression patterns in general it represses the mean of the intermediate genes, since more Edn1 does not induce the intermediate genes more strongly but lack of Edn1 leads to a loss of the intermediate domain, (see Fig 4). As a result the ventral domain expands and this effect is strongest when the I genes are expressed last (center).
(TIF)

S11 Fig Effects of combined noise in both morphogens and the GRN. 1D gene expression profiles for ventral (pink), intermediate (blue) and dorsal (yellow) genes showing that if noise in both Bmp and Edn1 gradients are present together with noise in the GRN. The thick lines show the mean value over 100 simulations and the shaded area is ±σ. The effects of the individual sources of noise are additive and noise in GRN dominates such that patterning the I domain last leads to the least precision in gene expression (center).
(TIF)

S12 Fig 2D modeling of patterning by extrinsic control of timing. (A) Control parameters if the temporal control is orchestrated by extrinsic factors, modeled by if-statements instead of intrinsic (modeled by varying production/degradation rates). (B) Final patterning with extrinsic temporal control, where the intermediate genes are expressed from the start of the simulation time (22 hpf), the ventral genes are only expressed after 24 hpf and the dorsal genes are turned on last, after 26 hpf.
(TIF)

S13 Fig Comparison between intrinsic (varying production/degradation rates) and extrinsic (if-statements) temporal control. 1D gene expression profiles for ventral (pink), intermediate (blue) and dorsal (yellow) genes, where the thick lines show the mean value over 100 simulations and the shaded area is ±σ. The simulations show that the gene expression profiles for extrinsic regulation of temporal patterning are less distinct than that of intrinsic regulation. Dorsal genes are expressed homogeneously across the domain. The effect of noise is similar to that of the minimal intrinsic model, where Bmp fluctuations have a stronger effect than those of Edn1 and the combined noise is dominated by noise in the GRN.
(TIF)

S14 Fig Bmp noise with extrinsic control. 1D gene expression profiles for ventral (pink), intermediate (blue) and dorsal (yellow) genes, where the thick lines show the mean value over 100 simulations and the shaded area is ±σ. With noise in the Bmp gradient, η1 = 1, and extrinsic control of gene expression timing the different sequences of D-V domain formation do not lead to different sensitivities to noise (in contrast to intrinsic timing, see Fig 7).
(TIF)

S15 Fig Comparison of the boundary accuracy with intrinsic and extrinsic temporal control. Statistics are collected from 10 simulations of the two-dimensional model, the line indicates the mean value and the error bars ±σ. If the timing is controlled intrinsically (varying production/degradation rates, red) the boundaries are more accurately positioned for individual sources of noise than when external factors control the temporal order (if-statements, black). However, the noise appears to be less additive with external control, such that for all sources of noise being present simultaneously the extrinsic model succeeds in more accurate positioning of the boundaries.
(TIF)

S1 Movie 1D model of arch patterning. Deterministic simulation of the 1D model of the mandibular arch along the D-V axis. A region is defined as patterned by a certain gene group if the expression level is above 20% of the maximum achieved over time (indicated by the black dashed line) and if expression is higher than that of the other gene groups. White regions are not yet expressing any genes. The narrow ventral domain corresponds to the thin layer of cells expressing ventral genes. Pink: ventral, cyan: intermediate, yellow: dorsal.
(MOV)

S2 Movie Morphogen gradients during patterning. A 2D simulation of arch patterning indicating the zone of production of both morphogens (green dots) and the resulting morphogen gradients are in grayscale. Patterning is further explained in S3 Movie.
(MOV)

S3 Movie 2D model of arch patterning. Deterministic simulation of the 2D model combining D-V and A-P axes. Here a single cell is defined as patterned (indicated by the corresponding color) if the gene expression level exceeds 20% of the maximum and is higher than the expression of the other genes. Grey indicates cells that are not yet expressing any genes above the 20% cut-off. Colors become deeper gradually as the level of expression increases. Pink: ventral, cyan: intermediate, yellow: dorsal, grey: unpatterned.
(MOV)

S4 Movie Live imaging of *hand2* transgene expression. Maximum projections of confocal imaging of live *hand2*:*eGFP;sox10*:*lyn-tdTomato* double-transgenic embryos. *hand2*:*eGFP* (green) is only expressed in ~2 ventral-most rows of cells, marking the ventral domain of the arches. *sox10*:*lyn-tdTomato* (red) marks all neural crest-derived (including arch) cells. *hand2*:*eGFP* is also expressed in a population of non-arch cells that overlap the arch in a maximum projection. The anterior of the embryo is to the left. Pharyngeal arches 1 and 2 are in the center of the image, and yolk autofluorescence is visible in the lower right.
(AVI)

S5 Movie Live imaging of *dlx5a* transgene expression. Maximum projections of confocal imaging of live *dlx5a*:*eGFP;sox10*:*lyn-tdTomato* double-transgenic embryos. *dlx5a*:*eGFP* (green) is expressed in ~4–5 rows of cells from the ventral border of the arch, marking the intermediate-ventral domain of the arches. Unlike mRNA expression (Fig 2), transgenic eGFP perdures in the ventral domain at later time points. *sox10*:*lyn-tdTomato* (red) marks all neural crest-derived (including arch) cells. The anterior of the embryo is to the left. Pharyngeal arches 1 and 2 are in the center of the image, and yolk autofluorescence is visible in the lower right. In this embryo, the arches undergo a pronounced compaction and rotation at later time points.
(AVI)

S6 Movie Stochastic 1D model of arch patterning. A single stochastic simulation from the 1D model of arch D-V patterning with GRN, Bmp, and Edn1 noise. Fig 6B–6E are derived from combined statistics of 100 of these individual simulations. Pink: ventral, cyan: intermediate, yellow: dorsal.
(MOV)

S7 Movie Stochastic 2D model of arch patterning. A single stochastic simulation from the 2D model of arch D-V patterning with GRN, Bmp, and Edn1 noise. When all three sources of noise are present simultaneously the effects are additive and the I domain is nearly lost while all three gene groups show strong fluctuations in their expression profiles as with noise only in the GRN.
(MOV)
