# Supplementary material for: Modeling craniofacial development reveals spatiotemporal constraints on robust patterning of the mandibular arch
Source: PLoS Comput Biol. 2018 Nov 27;14(11):e1006569. doi: 10.1371/journal.pcbi.1006569 (PMC6258504; doi:10.1371/journal.pcbi.1006569)
Supplement: S1 Table — The microscopic dissociation constants pi used to generate the mandibular gene regulatory network (GRN) for dorsal-ventral (D-V) patterning and the literature references on which the interactions are based (see Fig 1A and Eqs 1–3). (DOCX) [file pcbi.1006569.s001.docx]

| Interaction | | Value | Reference |
| --- | --- | --- | --- |
| p_1_ | Bmp promotes ventral identity | 0.0044 | Loss of Bmp signaling reduces *hand2* expression [24]  Increase in Bmp signaling increases *hand2* expression [24]  Increase in Bmp signaling increases *hand2* expression [30] |
| p_2_ | Edn1 promotes intermediate identity | 0.0005 | Loss of Edn signaling reduces *dlx3b* expression [27]  Loss of Edn1 signaling reduces *dlx3b*/*5a*/*6a* expression [29]  Increase in Edn1 signaling increases *dlx3b* and *dlx5a* expression [24]  Loss of Edn signaling reduces *dlx3b* expression [24] |
| p_3_ | Ventral identity inhibits intermediate analysis | 0.5 | Loss of *hand2* increases *dlx3b*/*4a*/*4b* expression [42]  Reduction of *hand2* increases *dlx3b* and *dlx5a* expression [30] |
| p_4_ | Dorsal identity inhibits intermediate identity | 0.7 | Loss of Jag/Notch signaling increases *dlx3b* expression [29]  Increase in Jag/Notch signaling reduces *dlx3b* expression [29] |
| p_5_ | Bmp promotes intermediate identity | 0.003 | Loss of Bmp signaling reduces *dlx3b* and *dlx6a* expression [24]  Increase in Bmp signaling increases *dlx3b* and *dlx5a* expression when *hand2* function is reduced [30] |
| p_6_ | Intermediate identity inhibits dorsal identity | 0.1 | Reduction of *dlx3b*/*4b*/*5a* increases the domain of *eng2* expression [42] |
| p_7_ | Edn1 inhibits dorsal identity | 0.01 | Loss of Edn1 signaling increases *jag1b* and *hey1* expression [29]  Increase in Edn1 signaling decreases *jag1b* and *hey1* expression [30] |
