# Supplementary material for: Modeling craniofacial development reveals spatiotemporal constraints on robust patterning of the mandibular arch
Source: PLoS Comput Biol. 2018 Nov 27;14(11):e1006569. doi: 10.1371/journal.pcbi.1006569 (PMC6258504; doi:10.1371/journal.pcbi.1006569)
Supplement: S2 Table — Production/degradation rates for the ventral (ve) intermediate (in) and dorsal (do) genes. One value is given since the production rate equals the degradation rate for each gene group. (DOCX) [file pcbi.1006569.s002.docx]

| Production and degradation parameters | | | | | | |
| --- | --- | --- | --- | --- | --- | --- |
|  | VID | VDI | IVD | IDV | DIV | DVI |
| *d_ve_* | 4.0 | 4.0 | 1.0 | 0.75 | 0.75 | 1.0 |
| *d_in_* | 1.0 | 0.25 | 4.0 | 4.0 | 1.0 | 0.25 |
| *d_do_* | 0.25 | 1.0 | 0.25 | 1.0 | 4.0 | 4.0 |
