# Supplementary material for: Modeling craniofacial development reveals spatiotemporal constraints on robust patterning of the mandibular arch
Source: PLoS Comput Biol. 2018 Nov 27;14(11):e1006569. doi: 10.1371/journal.pcbi.1006569 (PMC6258504; doi:10.1371/journal.pcbi.1006569)
Supplement: S3 Table — Parameters used to model mechanical forces between cells, between cells and surrounding tissues, and chemoattraction. (DOCX) [file pcbi.1006569.s003.docx]

| Mechanical parameters | | | | | |
| --- | --- | --- | --- | --- | --- |
| Intercellular | | Chemoattractant | | Boundary | |
| *U* | 200 | *W_1_* | 40000 | *S* | 100 |
| *V* | 1 | *W_2_* | 18000 | *ξ_5_* | 1 |
| *ξ_1_* | 1.25 | *ξ_3_* | 0.0001 |  | |
| *ξ_2_* | 2 | *ξ_4_* | 0.0001 |  |  |
|  | | *s_1_* | 200 |  |  |
|  |  | *s_2_* | -350 |  |  |
