# Supplementary figures and images for: Modeling craniofacial development reveals spatiotemporal constraints on robust patterning of the mandibular arch
Source: PLoS Comput Biol. 2018 Nov 27;14(11):e1006569. doi: 10.1371/journal.pcbi.1006569 (PMC6258504; doi:10.1371/journal.pcbi.1006569)

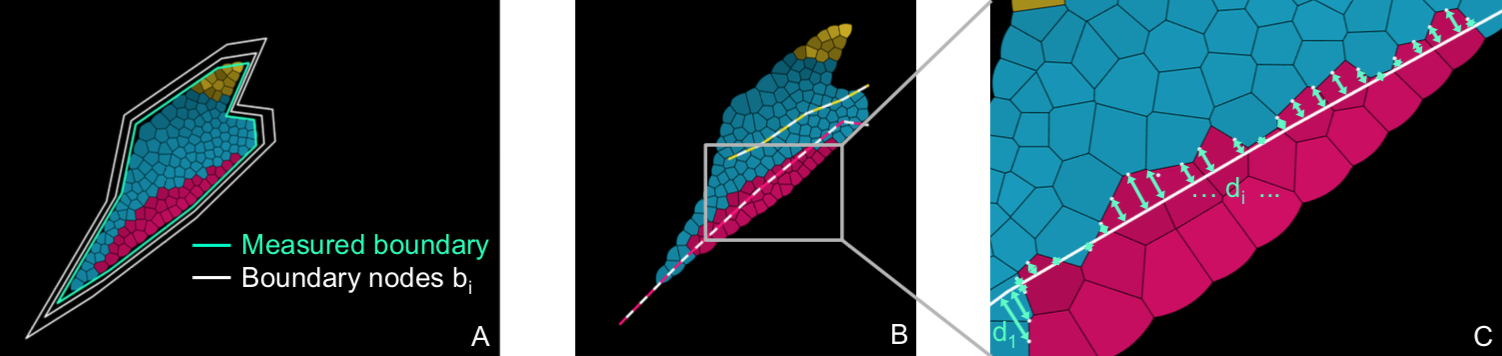

Supplement: S1 Fig — (A) Simulation results of the two-dimensional (2D) model with the measured boundary (green line), averaged over 6 sets of zebrafish images. To enforce the simulated cells to stay inside the measured arch outlines (green line) we put two rings of a total of 2118 boundary nodes around the outline (white lines). These nodes exert a repulsive force (Eq 7) on the cell centers xi, such that the cells do not leave the green perimeter. (B) Simulation results of the 2D model overlayed with the domain boundaries averaged over three sets of images from zebrafish embryos for the V-I (pink-white dashed line) and I-D (yellow-white dashed line) boundaries. (C) Boundary error E is the sum of the distances di (green arrows) between the simulated domain boundary for each cell at the boundary (white dots) and the actual measured boundary (white line). (TIF) [file pcbi.1006569.s004.tif]

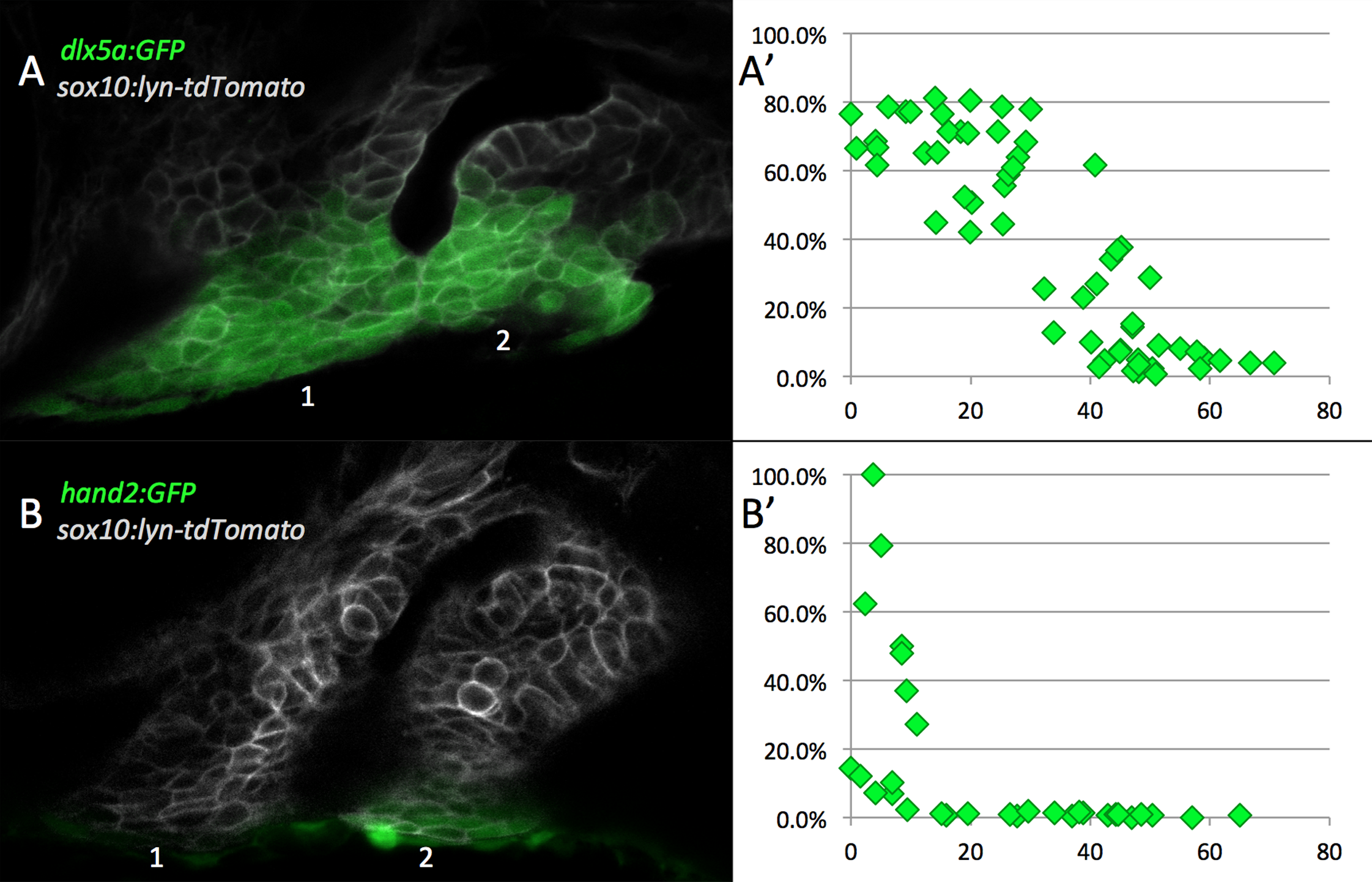

Supplement: S2 Fig — Single confocal z-slices of dlx5:GFP;sox10:lyn-tdTomato (A) and hand2:GFP;sox10:lyn-tdTomato (B) double-transgenic embryos at 30 hpf. For both the intermediate-dorsal boundary (A) and ventral-intermediate boundary (B), quantification of per-cell fluorescence intensity in arches 1 and 2 reveals two distinct populations of cells, those with high signal intensity and those with low signal intensity, indicating an abrupt drop-off in fluorescence signal and thus a sharp boundary of transgene expression. In the graphs, the y-axis shows mean GFP intensity per cell, normalized to the maximum possible intensity. The x-axis shows the D-V position of each cell, measured in μm with the ventral edge of the arch at 0. (TIF) [file pcbi.1006569.s005.tif]

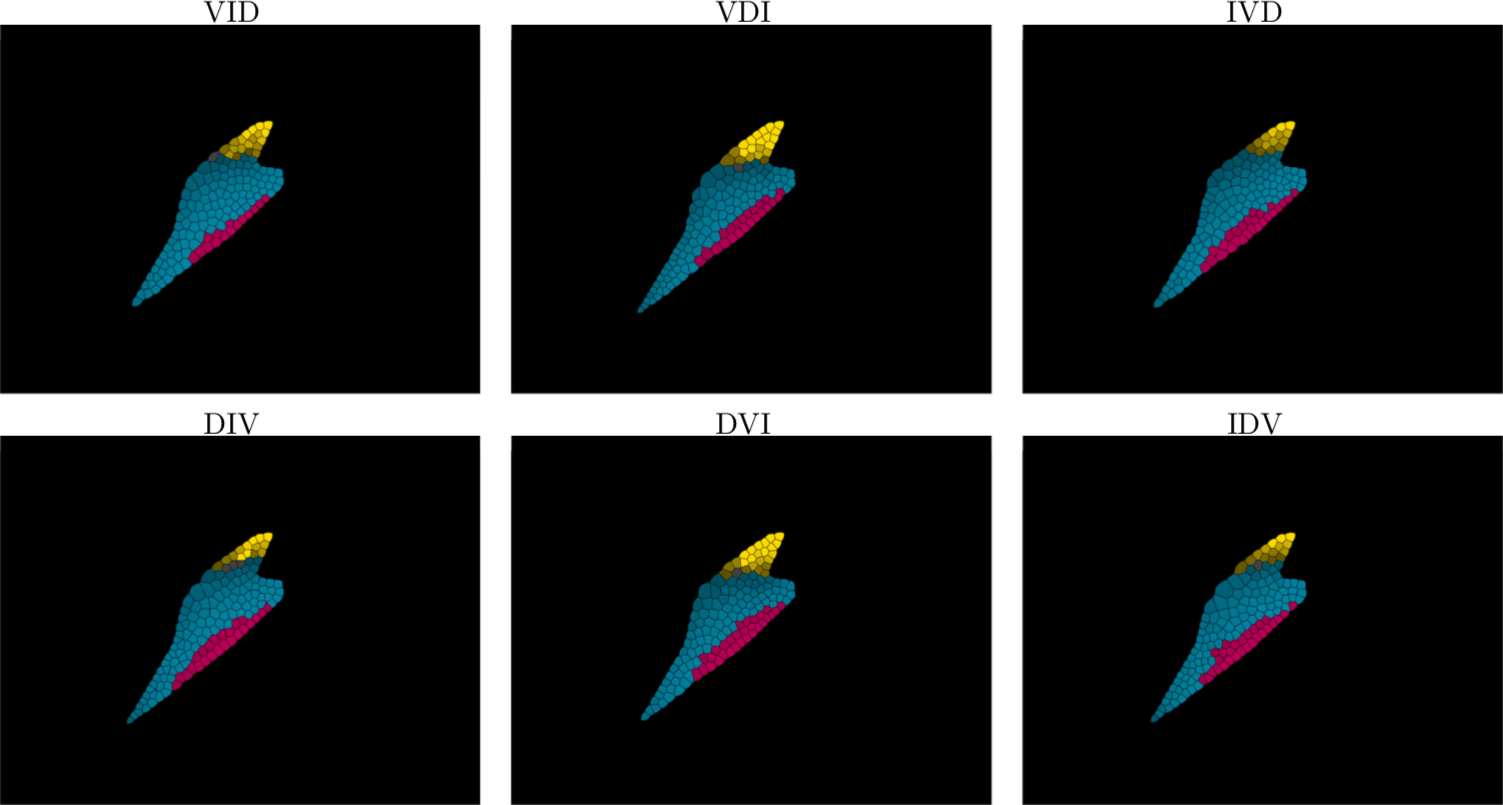

Supplement: S3 Fig — By simply switching the absolute values of the production/degradation rates while keeping their ratio constant (see S1 Table), the three gene groups (dorsal, intermediate and ventral) can be expressed in any of the 6 possible orders and still lead to a correct final pattern at 35 hpf. (TIF) [file pcbi.1006569.s006.tif]

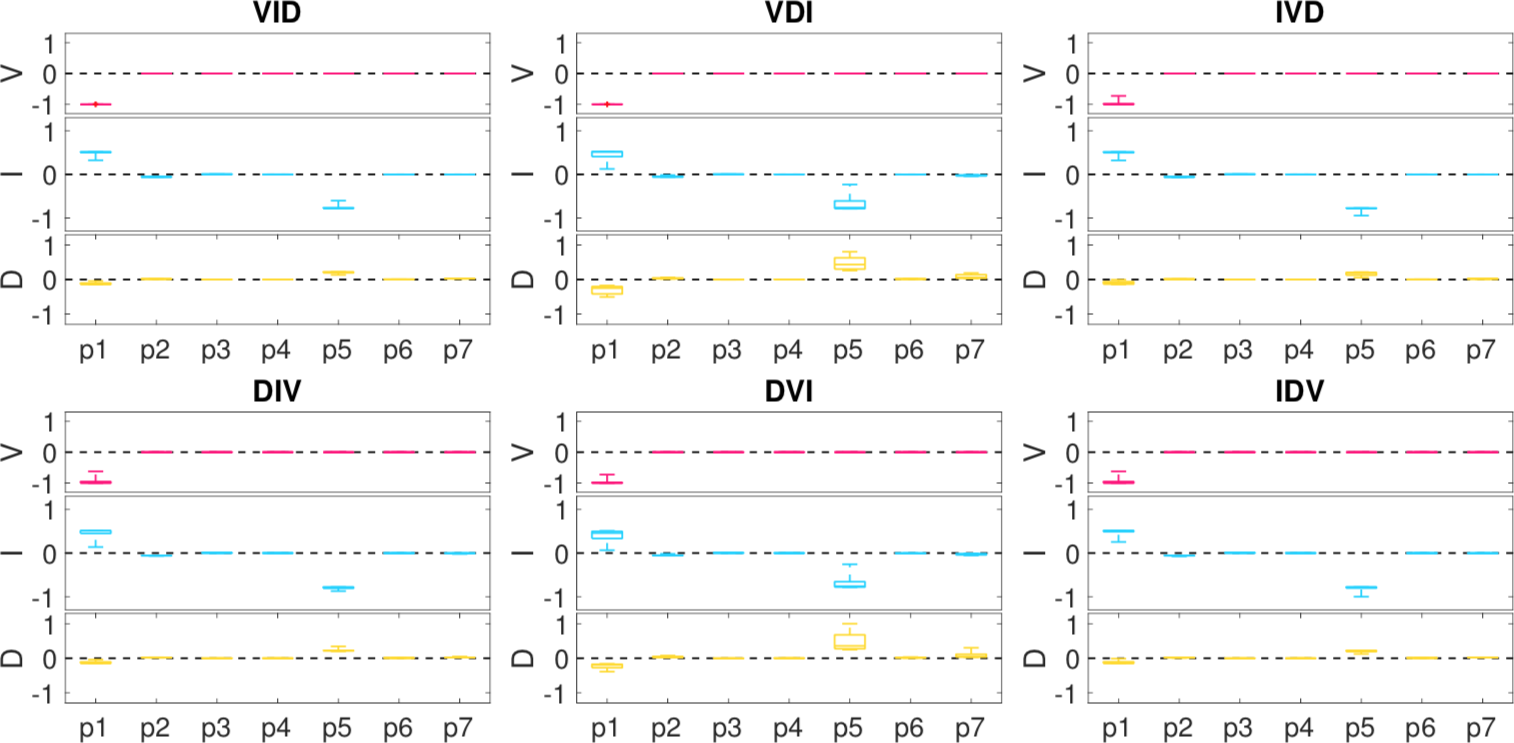

Supplement: S4 Fig — Sensitivity plots for all gene groups with respect to the parameters p1−p7 (see Fig 1A), when cells experience morphogen concentrations typical of the V domain. The values are normalized with respect to the strongest sensitivity over time in all of the parameters for each gene V,I and D individually. The Bmp parameters p1 and p5 are most sensitive to fluctuations (see Fig 5). The temporal order mostly effects the sensitivity of the dorsal domain, where expressing I last (center) leads to the strongest sensitivity of D. (TIF) [file pcbi.1006569.s007.tif]

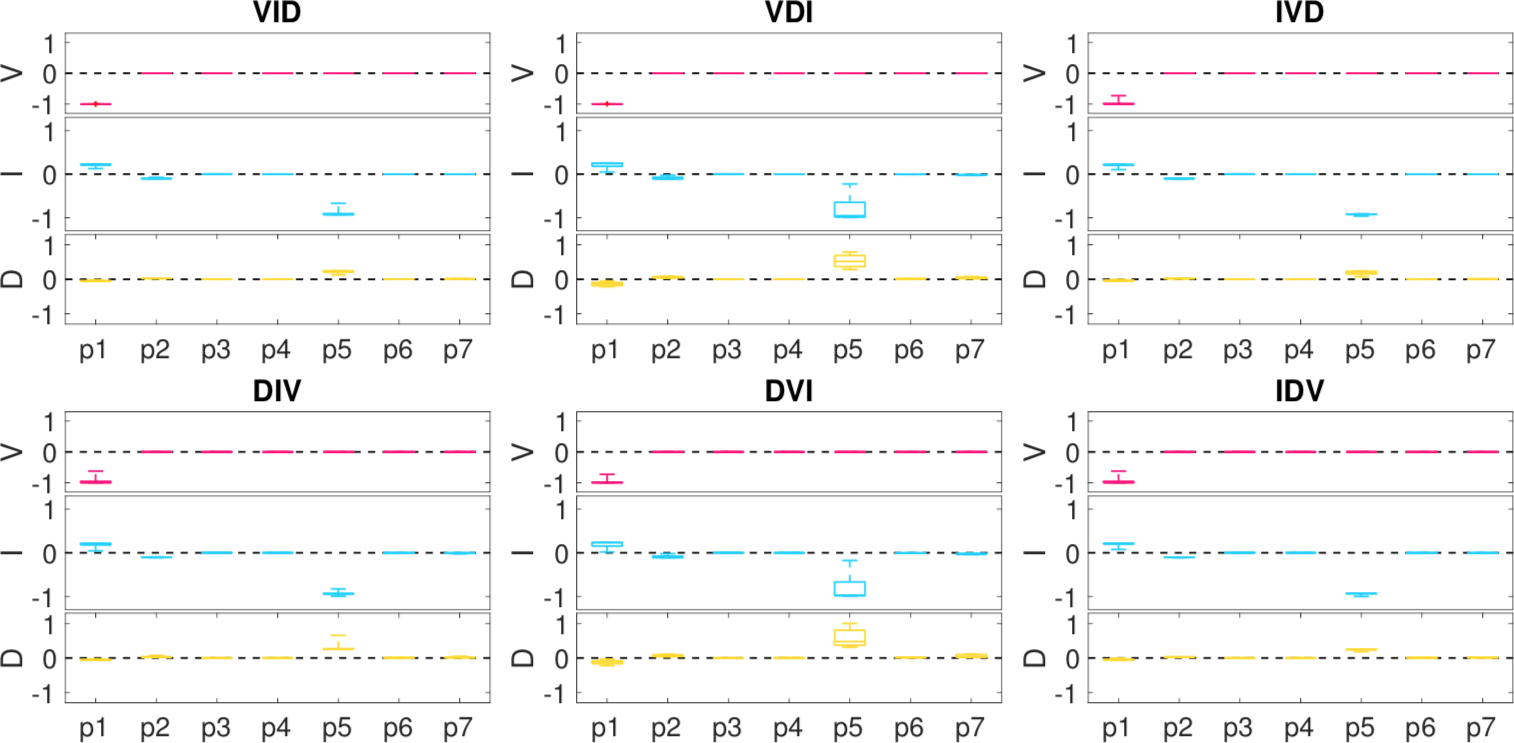

Supplement: S5 Fig — Sensitivity plots for all gene groups with respect to the parameters p1 − p7 when cells experience morphogen concentrations typical of those in the I domain. The values are normalized with respect to the strongest sensitivity over time in all of the parameters for each gene V, I and D individually. The GRN is again most sensitive to perturbations in the parameters modeling the BMP effect (p1 and p5), though the sensitivity of I and D with respect to p1 has been reduced compared to the ventral domain, due to the lower concentration of Bmp. The temporal order is again mostly significant in the dorsal domain, where expressing I last (center) leads to the strongest sensitivity of D. (TIF) [file pcbi.1006569.s008.tif]

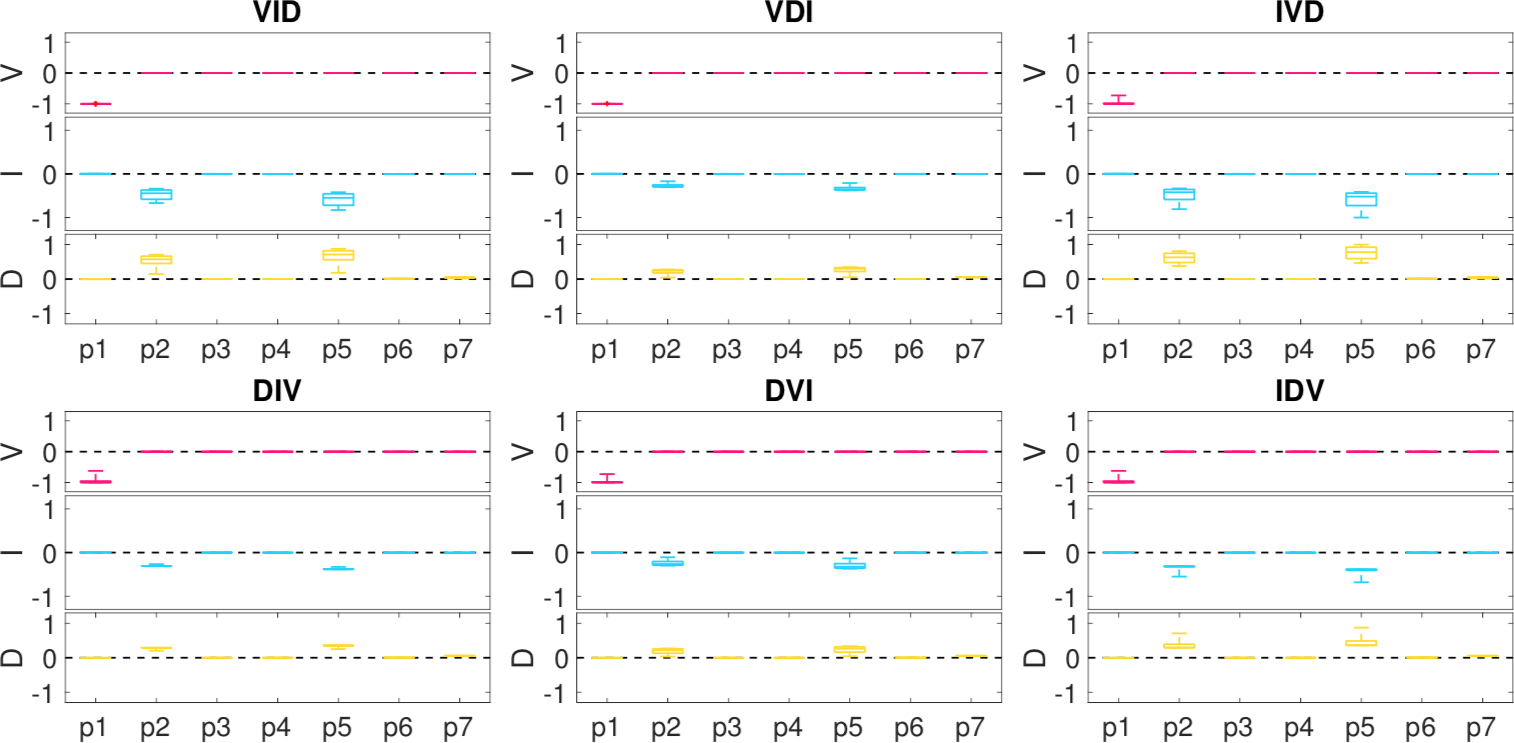

Supplement: S6 Fig — Sensitivity plots for all gene groups with respect to the parameters p1 − p7, when cells experience morphogen concentrations typical to those in the D domain. The values are normalized with respect to the strongest sensitivity over time in all of the parameters for each gene V, I and D individually. Since both the Bmp and ventral gene concentrations are very low in the dorsal domain, the intermediate gene expression is here most sensitive to the direct control by the two morphogens (Edn1 (p2) and Bmp (p5), see Fig 1A). Similarly the dorsal gene expression depends on those two parameters by indirect interaction through the intermediate genes. The temporal order here favors a late expression of intermediate genes (center). (TIF) [file pcbi.1006569.s009.tif]

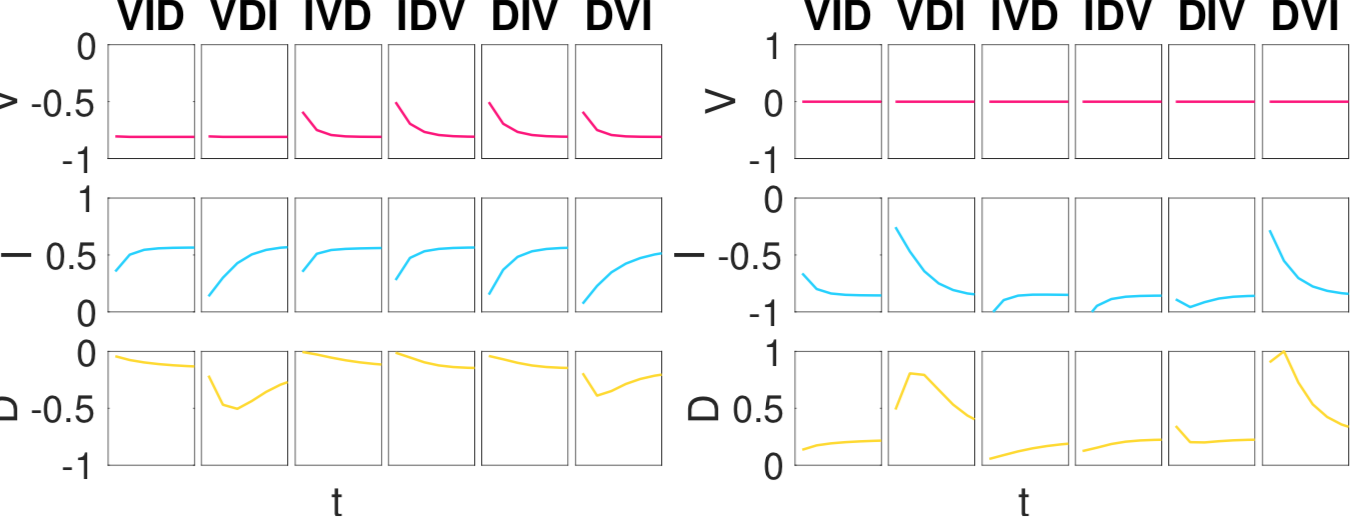

Supplement: S7 Fig — The normalized sensitivities si(t) plotted over time (x-axis) for genes expressed in the D, I and V domains with respect to the parameters p1 (left plots) and p5 (right plots). These parameters both model the influences of BMP (see Fig 1A) and are the most sensitive to the domain order. The sensitivity to both parameters p1 and p5 deviates the most from zero at early times, when genes in the I domain are expressed last (VDI and DVI). (TIF) [file pcbi.1006569.s010.tif]

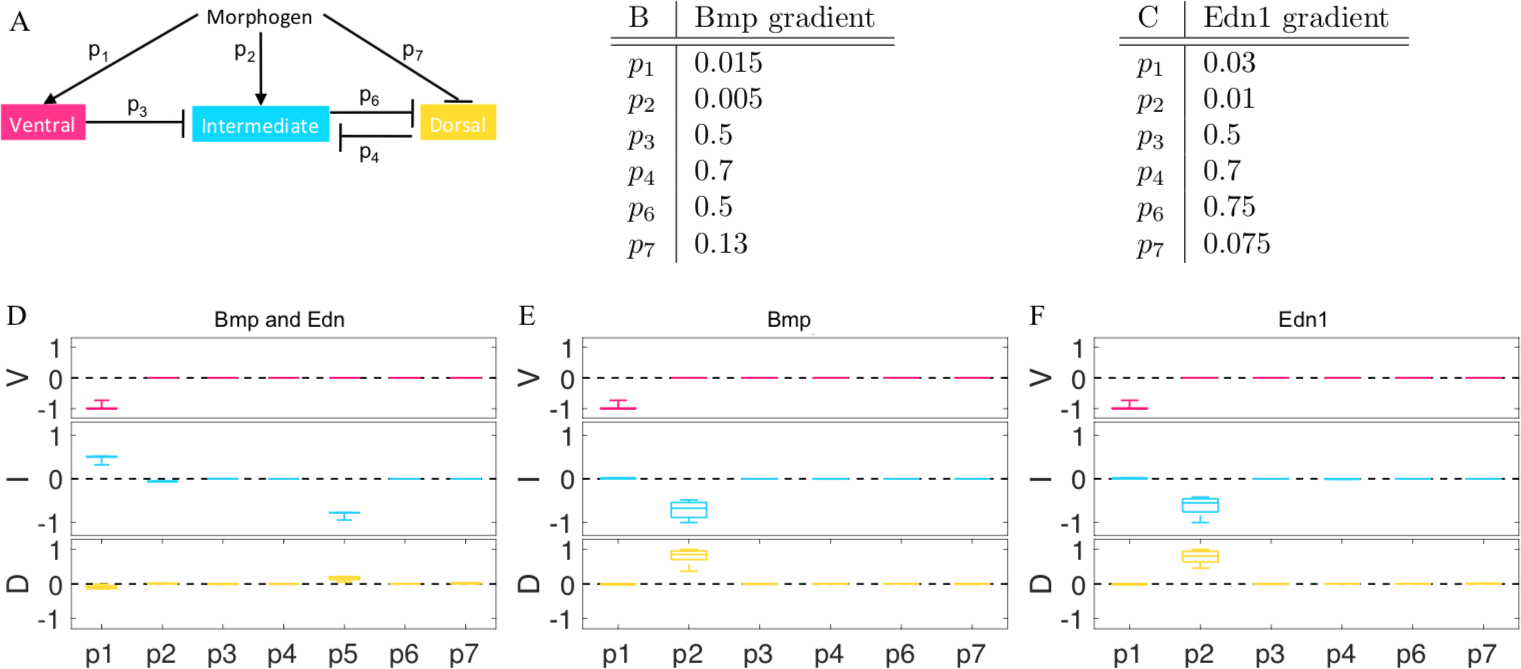

Supplement: S8 Fig — (A) The GRN used to compute gene expression inside each cell if only one D-V morphogen gradient emanating from ventral is controlling the patterning. The parameters p2 and p5 in Fig 1A merge into only p2 here. (B,C) The control parameters pi when the single morphogen gradients is either Bmp (short range) or Edn1 (long range). (D) The sensitivities in a two morphogen GRN for comparison with the IVD order and morphogen concentrations typical for the ventral domain (see also S4 Fig, IVD). (E, F) The sensitivity of the three gene groups when only one gradient with extent similar to Bmp or Edn1, respectively controls the GRN. The parameter p1 is still the only one with effect on ventral gene expression and p2 now accumulates the sensitivity of the intermediate and dorsal genes compared to p2 and p5 in the two morphogen model. (TIF) [file pcbi.1006569.s011.tif]

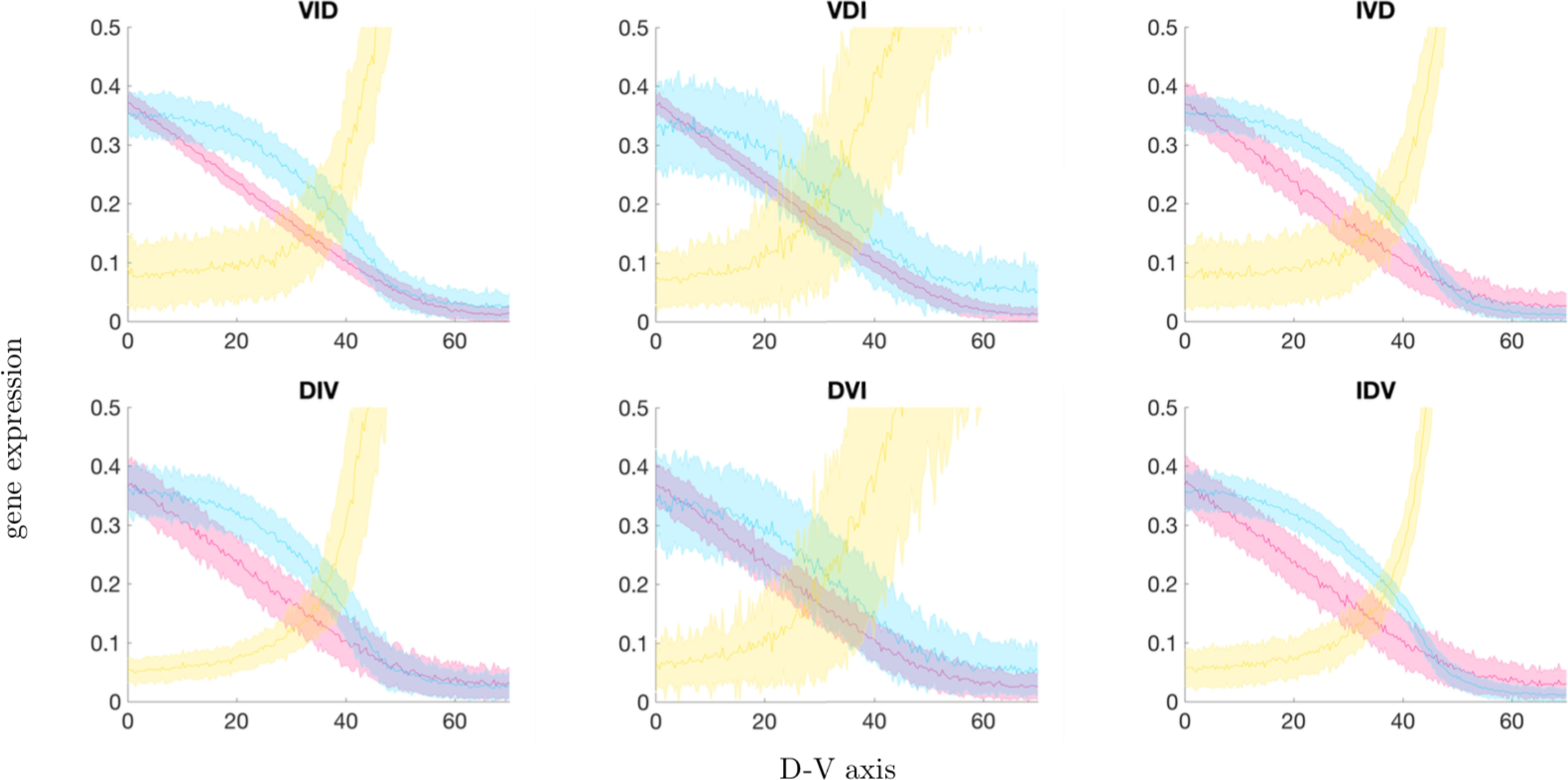

Supplement: S9 Fig — 1D gene expression profiles for ventral (pink), intermediate (blue) and dorsal (yellow) genes showing that with noise in the GRN, ν = 0.05. The thick lines show the mean value over 100 simulations and the shaded area is ±σ. Genes expressed earliest are more robust and less susceptible to noise than genes expressed later. Especially in the case when I is expressed last (center) the GRN is most susceptible to noise, in agreement with the findings in S4 Fig and S5 Fig. (TIF) [file pcbi.1006569.s012.tif]

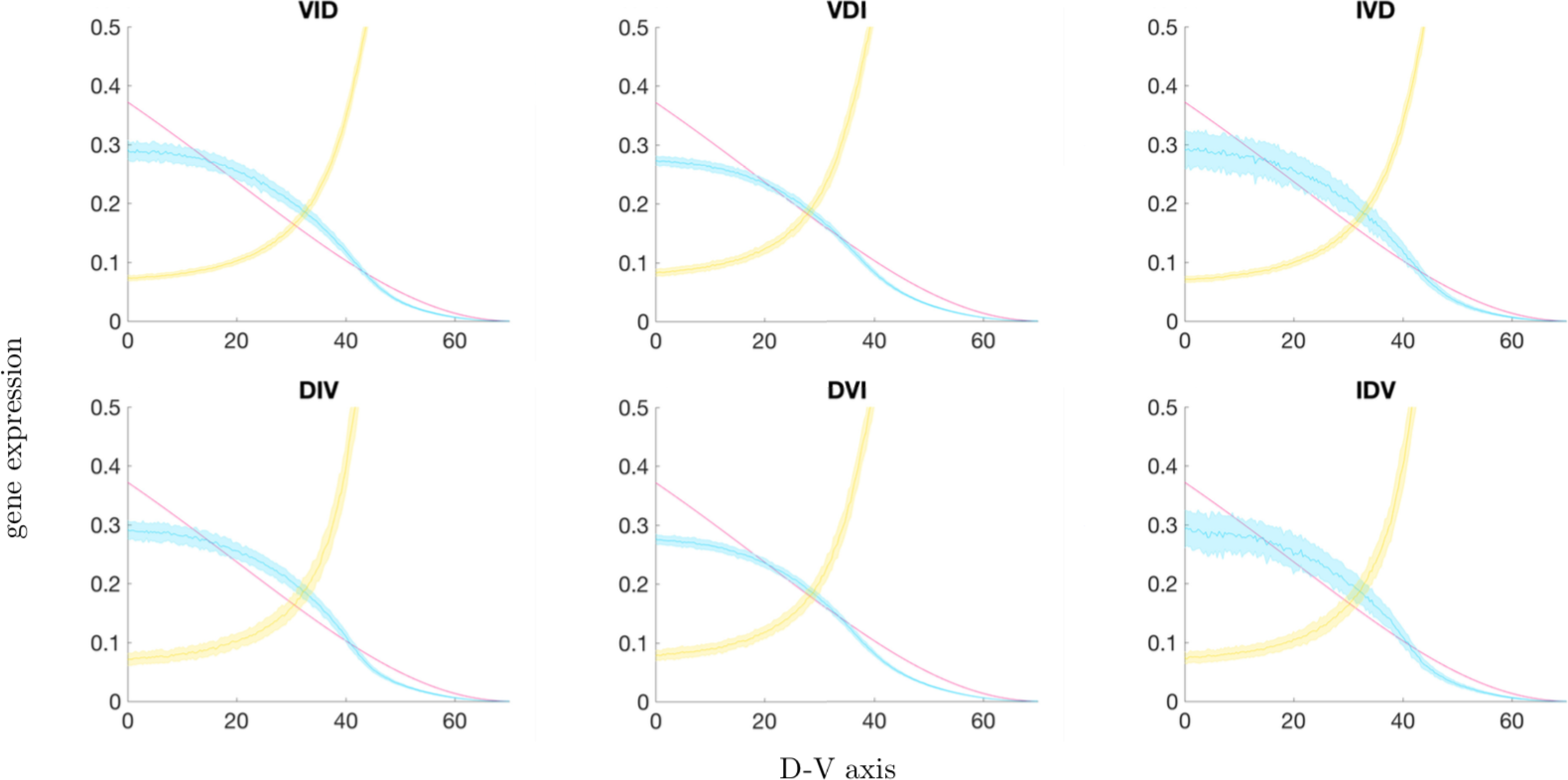

Supplement: S10 Fig — 1D gene expression profiles for ventral (pink), intermediate (blue) and dorsal (yellow) genes with noise in Edn1, η2 = 1. The thick lines show the mean value over 100 simulations and the shaded area is ±σ. The simulations show that Edn1 fluctuations affect the intermediate gene group most strongly, especially when I is expressed first (right). Although Edn1 noise has a smaller effect on the deviation in expression patterns in general it represses the mean of the intermediate genes, since more Edn1 does not induce the intermediate genes more strongly but lack of Edn1 leads to a loss of the intermediate domain, (see Fig 4). As a result the ventral domain expands and this effect is strongest when the I genes are expressed last (center). (TIF) [file pcbi.1006569.s013.tif]

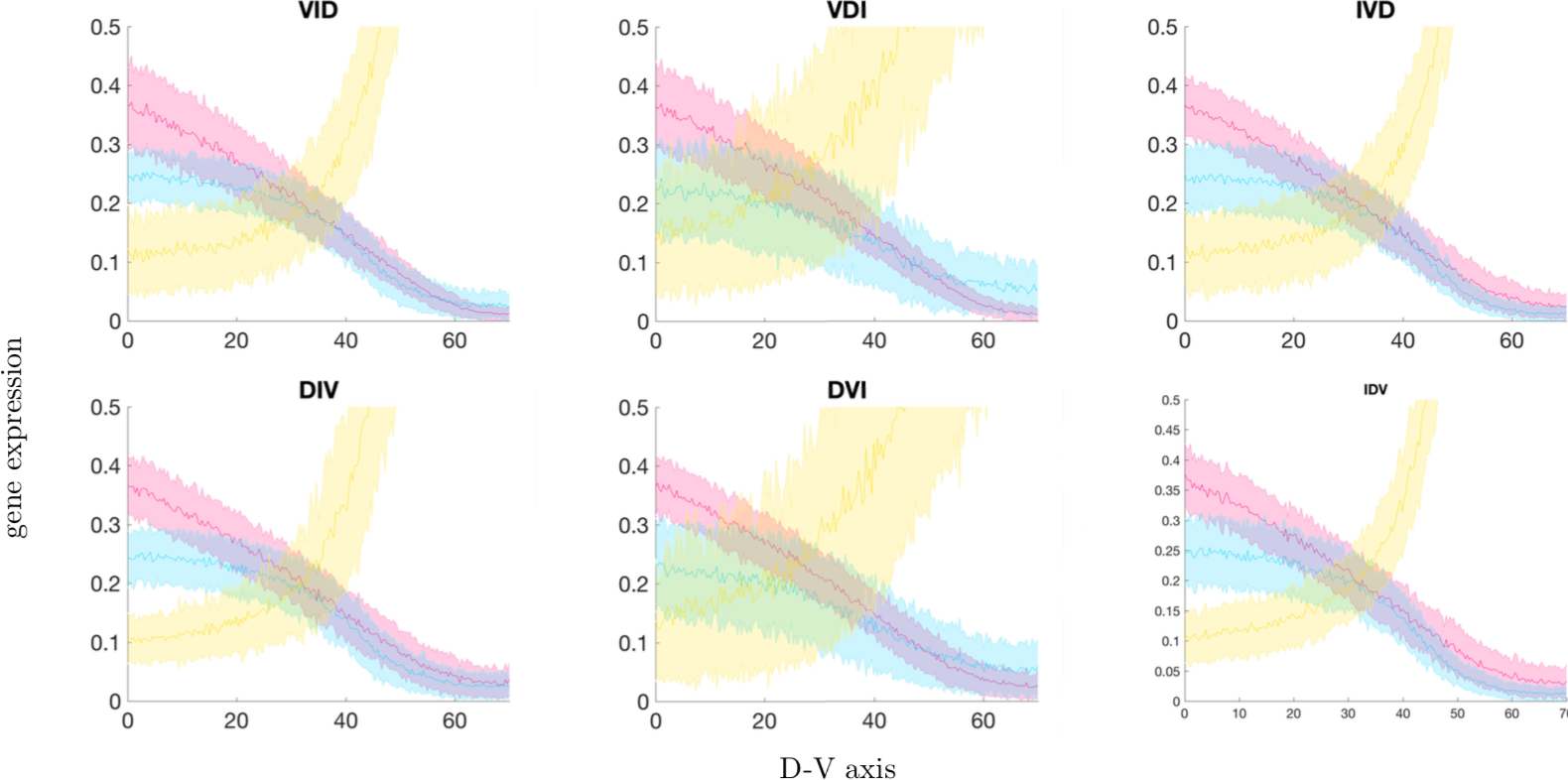

Supplement: S11 Fig — 1D gene expression profiles for ventral (pink), intermediate (blue) and dorsal (yellow) genes showing that if noise in both Bmp and Edn1 gradients are present together with noise in the GRN. The thick lines show the mean value over 100 simulations and the shaded area is ±σ. The effects of the individual sources of noise are additive and noise in GRN dominates such that patterning the I domain last leads to the least precision in gene expression (center). (TIF) [file pcbi.1006569.s014.tif]

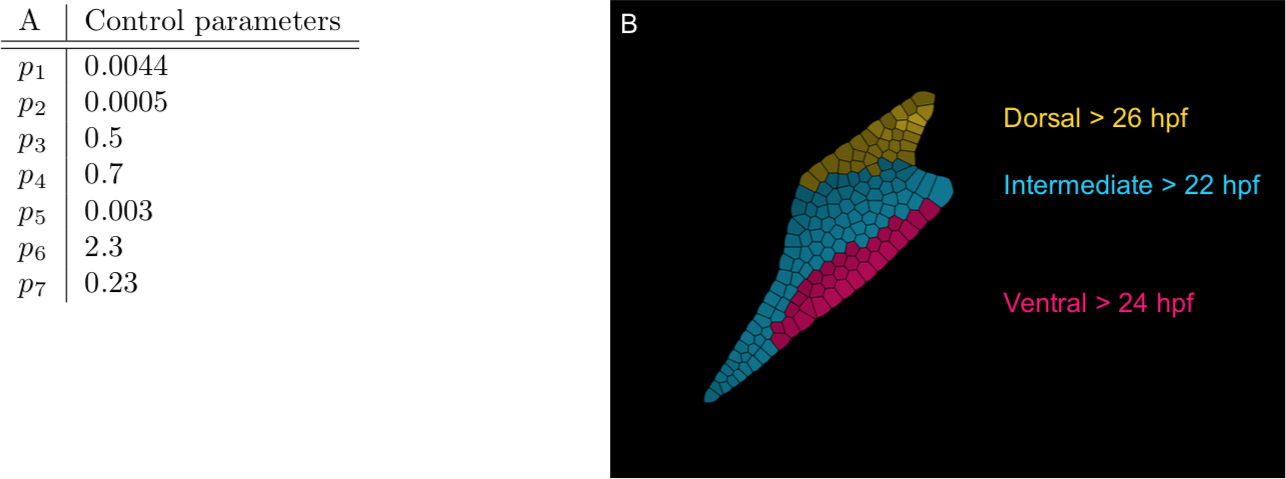

Supplement: S12 Fig — (A) Control parameters if the temporal control is orchestrated by extrinsic factors, modeled by if-statements instead of intrinsic (modeled by varying production/degradation rates). (B) Final patterning with extrinsic temporal control, where the intermediate genes are expressed from the start of the simulation time (22 hpf), the ventral genes are only expressed after 24 hpf and the dorsal genes are turned on last, after 26 hpf. (TIF) [file pcbi.1006569.s015.tif]

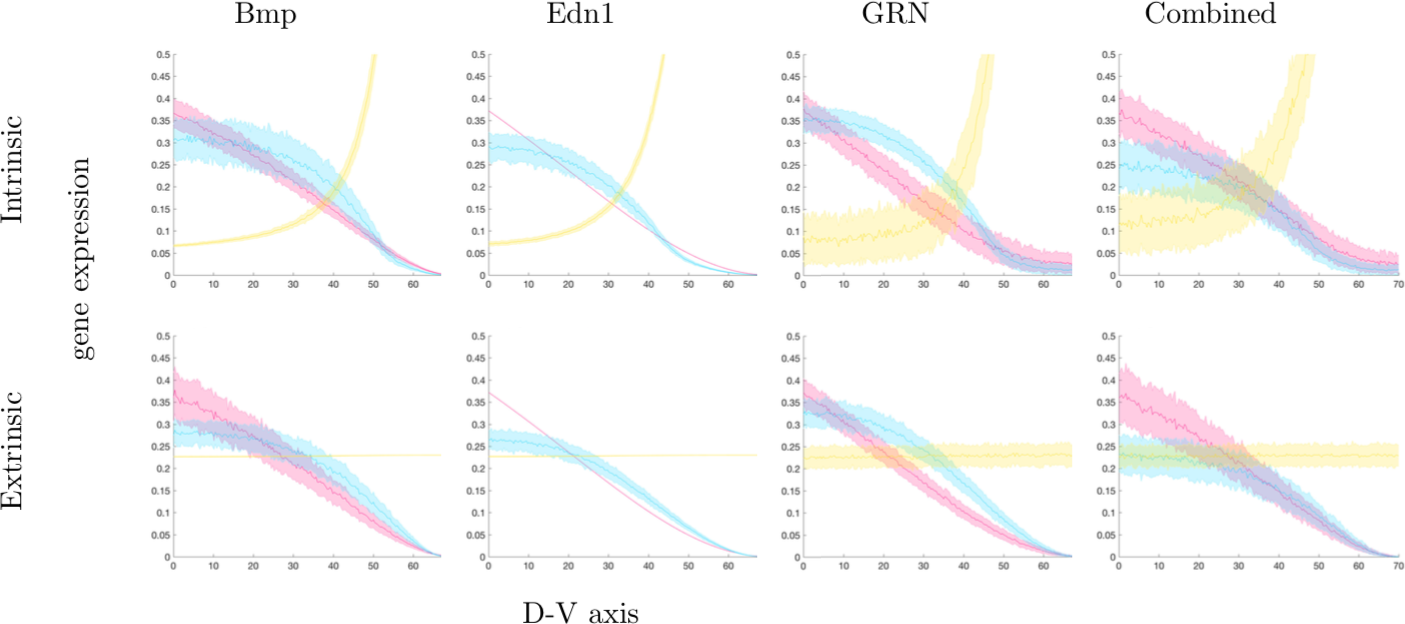

Supplement: S13 Fig — 1D gene expression profiles for ventral (pink), intermediate (blue) and dorsal (yellow) genes, where the thick lines show the mean value over 100 simulations and the shaded area is ±σ. The simulations show that the gene expression profiles for extrinsic regulation of temporal patterning are less distinct than that of intrinsic regulation. Dorsal genes are expressed homogeneously across the domain. The effect of noise is similar to that of the minimal intrinsic model, where Bmp fluctuations have a stronger effect than those of Edn1 and the combined noise is dominated by noise in the GRN. (TIF) [file pcbi.1006569.s016.tif]

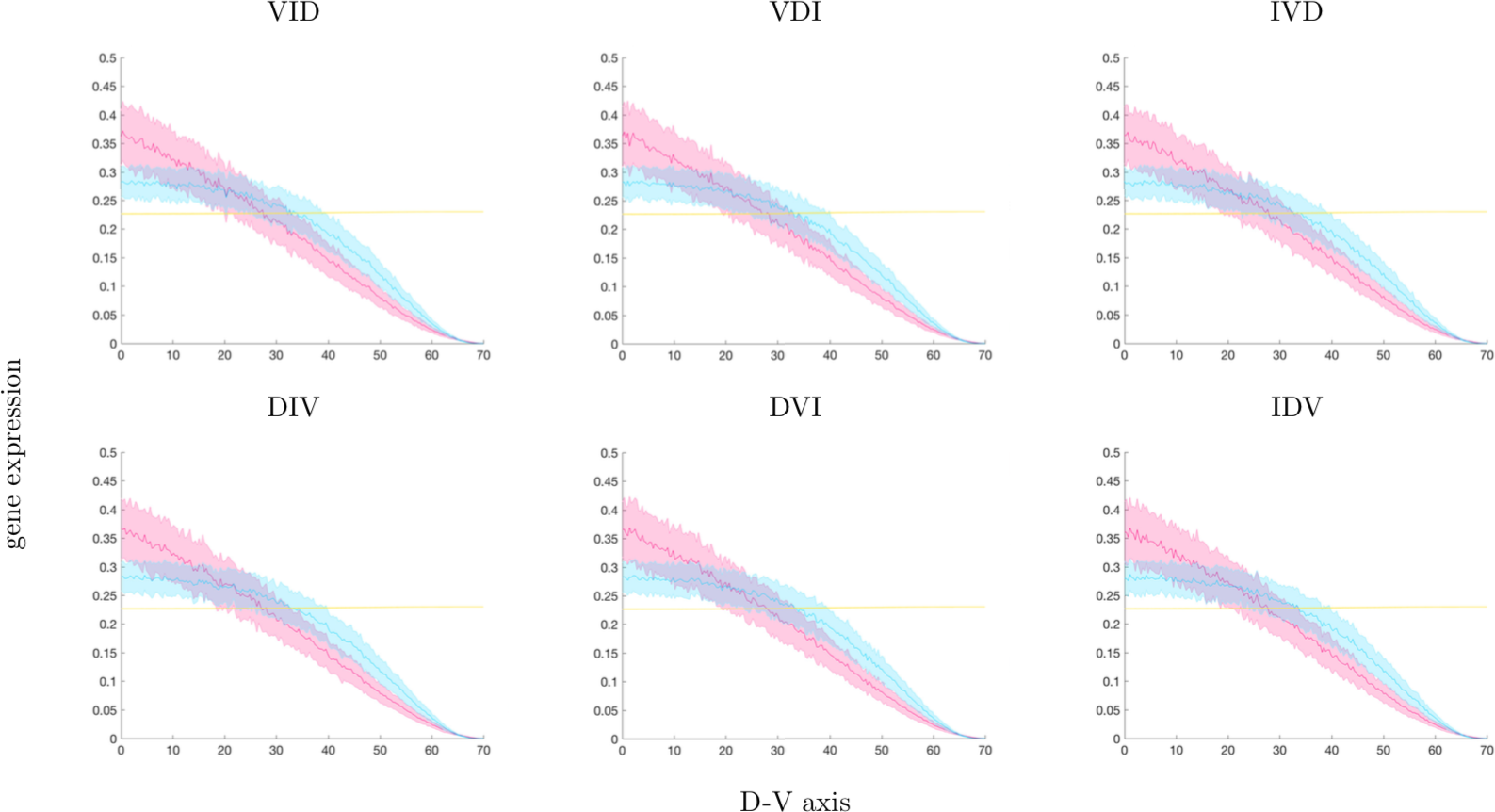

Supplement: S14 Fig — 1D gene expression profiles for ventral (pink), intermediate (blue) and dorsal (yellow) genes, where the thick lines show the mean value over 100 simulations and the shaded area is ±σ. With noise in the Bmp gradient, η1 = 1, and extrinsic control of gene expression timing the different sequences of D-V domain formation do not lead to different sensitivities to noise (in contrast to intrinsic timing, see Fig 7). (TIF) [file pcbi.1006569.s017.tif]

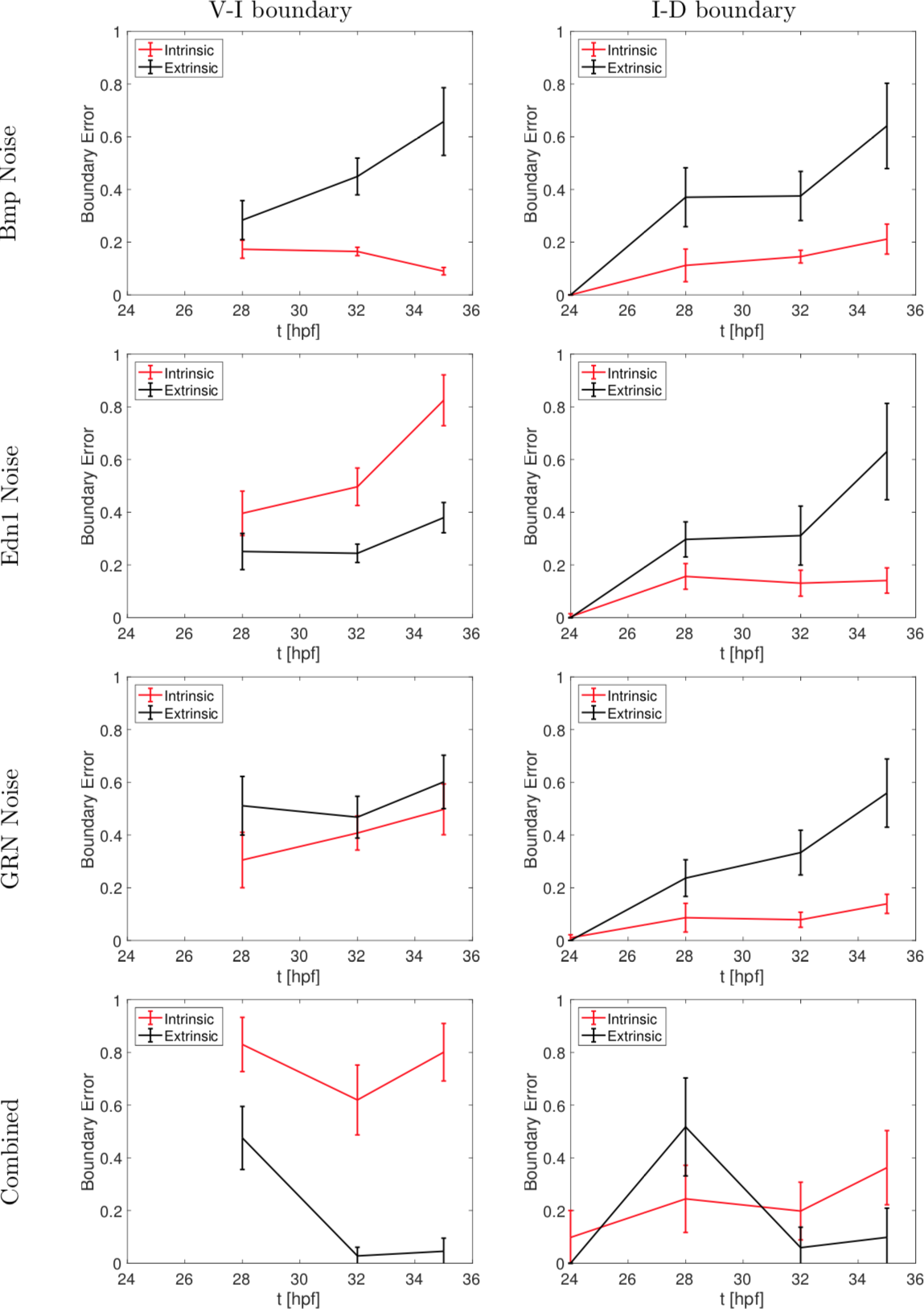

Supplement: S15 Fig — Statistics are collected from 10 simulations of the two-dimensional model, the line indicates the mean value and the error bars ±σ. If the timing is controlled intrinsically (varying production/degradation rates, red) the boundaries are more accurately positioned for individual sources of noise than when external factors control the temporal order (if-statements, black). However, the noise appears to be less additive with external control, such that for all sources of noise being present simultaneously the extrinsic model succeeds in more accurate positioning of the boundaries. (TIF) [file pcbi.1006569.s018.tif]
